# Polyherbal formulation PL02 alleviates pain, inflammation, and subchondral bone deterioration in an osteoarthritis rodent model

**DOI:** 10.3389/fnut.2023.1217051

**Published:** 2023-11-16

**Authors:** Prabhat Upadhyay, Diya Kalra, Aishwarya Shrikant Nilakhe, Vijay Aggrawal, Sarika Gupta

**Affiliations:** ^1^Molecular Science Lab, National Institute of Immunology (NII), New Delhi, India; ^2^Wellman Center for Photomedicine, Massachusetts General Hospital, Harvard Medical School, Boston, MA, United States; ^3^M/s Purobien Lifesciences Ltd, Baddi, Himachal Pradesh, India

**Keywords:** inflammation, monosodium iodoacetate, pro-inflammatory, chemokines, chondroprotective

## Abstract

**Introduction:**

Osteoarthritis (OA) is a debilitating disease with significant personal and socioeconomic burdens worldwide.

**Methods:**

To address this, we developed a multitargeted formulation called PL02, which includes standardized extracts of *Rosa canina* L, *Hippophae rhamnoides*, and collagen peptide. We tested the pharmacological efficacy of PL02 in a rodent model of OA induced by Monosodium iodoacetate (MIA).

**Results:**

Our results demonstrate that oral administration of PL02 has antioxidant effects by down-regulating NOS, reduces pain-related behavior, and mitigates inflammation by inhibiting IL-1b and TNF-α production, as well as downregulating CGRP1 and COX-II. PL02 also exhibits anti-catabolic and chondroprotective activity by significantly downregulating MMP13 and upregulating BCL2. Additionally, PL02 demonstrates chondrogenic activity by significantly upregulating SOX-9 (a master regulator of chondrogenesis), Coll-I, and aggrecan, which are major components of articular cartilage. Furthermore, PL02 prevents microarchitectural deterioration of subchondral bone.

**Conclusion:**

Overall, PL02 is an orally active, multi-targeted therapy that not only alleviates pain and inflammation but also effectively halts cartilage and subchondral bone deterioration. It represents a safe and promising candidate for the treatment and management of OA.

## 1 Introduction

Osteoarthritis (OA) is a chronic degenerative condition that leads to the deterioration of articular cartilage and deformation of subchondral bones, resulting in pain and significantly impacting more than 500 million people worldwide (1). This is a common condition among older individuals, characterized by the calcification of bones, loosening of ligaments surrounding joints, and resulting in chronic pain, joint stiffness, and inflammation. As the disease advances, it disrupts the homeostasis of cartilage and non-cartilaginous components of the joint, including the joint capsule, synovium, subchondral bone, ligaments, and periarticular muscles (2). The subchondral bone thickens, and the formation of osteophytes in OA joints leads to increased friction, inflammation in the synovium, and uneven enlargement of the joint (3). Several risk factors contribute to the development of OA, such as age, obesity, oxidative stress, inflammation, nutritional deficiencies, genetic factors, and trauma (4). Nonsteroidal anti-inflammatory drugs (NSAIDs) are commonly used as a first-line therapy for OA-related pain. However, their long-term efficacy declines, and they can cause significant adverse gastrointestinal and cardiovascular events. Currently, there is no effective therapy for OA, and the available options (herbal remedies, NSAIDs, and steroids) provide only short-term symptomatic relief and are poorly tolerated. Hence, there is an urgent need to develop effective and safe non-opioid treatments to prevent and resolve the pathophysiology of OA. Oxidative stress and inflammation play crucial roles in the development of OA, leading to cartilage degeneration and subchondral bone deformation, ultimately contributing to disease progression. Targeting excessive oxidative stress, inflammation, and promoting cartilage regeneration holds promise for alleviating chronic pain and improving articular cartilage to halt disease progression (5, 6).

Numerous research groups worldwide have investigated the pharmaceutical efficacy of various bioactive phytochemicals in the treatment of diverse human illnesses (7). One such example is Rosehips (*Rosa canina*-Scientific name), which are the aggregate fruits of shrubs belonging to the Rosa genus of the Rosaceae family. They are widely recognized for their antioxidant properties and are commonly used as dietary antioxidants (8). The high antioxidant activity is primarily attributed to ascorbic acid. Additionally, rosehips contain various other phytochemicals such as carotenoids, tocopherols, polyphenolic compounds, and triterpenoic acids, which have demonstrated immunomodulatory, hepatoprotective, anti-inflammatory, antitumor, antioxidant, and antihyperlipidemic effects in both *in vitro* and *in vivo* studies. In *in vitro* experiments, the unsaturated fatty acids, mainly linoleic and α-linolenic acids, present in rosehip seeds have shown inhibitory effects on cyclooxygenase-1 and 2 (9, 10)^.^

*Hippophae rhamnoides* (Seabuckthorn-common name), belonging to the family Elaeagnaceae, is another plant of significant importance, although less extensively studied. In India, *Hippophae rhamnoides* thrives in the high-temperate zones of the Western Himalayas, particularly in Leh, Laddakh, Himachal Pradesh, Uttarakhand, and Sikkim, at elevations ranging from 2,600 to 4,000 meters. This plant has been recognized for its beneficial properties, serving as a natural antioxidant with anti-inflammatory, immunomodulatory, and homocysteine-reducing effects (11). The phytochemicals present in *Hippophae rhamnoides* have demonstrated potential in preventing mitochondrial degeneration, reducing vascular inflammation, and exhibiting vasodilatory and anti-cancer activities. Furthermore, the evaluation of Seabuckthorn (SBT) leaf extract in an adjuvant-induced arthritis (AIA) rat model revealed significant anti-inflammatory activity, suggesting its potential for arthritis treatment (12, 13).

Collagen, the most abundant biomaterial in the body, is essential for the structure of cartilage, skin, and bone. Studies indicate that collagen levels decline by approximately 1% per year after the age of 25. Hydrolyzed collagen, which consists of small molecular weight peptides, has better bioavailability compared to whole collagen and gelatin. It is believed to have potential benefits for skin, bone, and joint health (14, 15). Supplementation with hydrolyzed collagen may aid in replenishing collagen levels that decline due to aging and an inadequate diet. It is thought to stimulate collagen production in the extracellular matrix of cartilage and other tissues. However, further research is needed to establish its therapeutic potential.

The MIA-induced OA model is widely recognized in the literature for its ability to replicate symptoms mimic to human OA. The injection of MIA into the joint induces an inflammatory response, with pain being the predominant symptom. The MIA injection leads to chondrocyte cell death, resulting in cartilage degeneration and subsequent alterations in the subchondral bone. This model serves as an important tool for studying the pathogenesis of OA (16–19).

Despite the known individual therapeutic potential of collagen peptide, Rosehip, and *Hippophae rhamnoides* as anti-inflammatory and immunomodulatory agents, they have not yet been considered as first-line therapy for pain and inflammation associated with osteoarthritis (OA) and other related diseases. In the present study, we aimed to address this gap by standardizing and characterizing the extracts of Rosehip and *Hippophae rhamnoides* and developing a formulation, named PL02, that incorporates these extracts along with collagen peptides. To evaluate the pharmacological effects of the formulation in managing OA, we utilized the MIA-induced animal model of OA. We assessed various parameters including the levels of anti-inflammatory markers, antioxidants, chemokines, cytokines, gene expression, conducted histological analysis of joint tissue, and analyzed the microarchitecture of subchondral bone. Through these evaluations, we aimed to decipher the mechanism of action of the formulation in managing OA.

## 2 Materials and methods

### 2.1 Materials and reagents

Monosodium iodoacetate (MIA), sodium chloride (NaCl), Sephadex G-15 beads were purchased from Sigma-Aldrich (St. Louis, United States). Plant extracts of Rosehip (*Rosa canina* L.) and Seabuckthorn (*Hippophae rhamnoides*) were a purchased from GMP certified company. Fish Collagen peptide was procured from FSSAI approved company. The XK 16/70 used for collagen desalting and purification was purchased from GE Healthcare Bio-Sciences AB (Uppsala, Sweden). Serum glutamic oxaloacetic transaminase (SGOT), Serum glutamic pyruvic transaminase (SGPT) Alkaline phosphatase (ALP), Urea, Creatinine kits for biochemical estimation were purchased from Tulip Diagnostics Private Limited. Lipid Peroxide estimation was done using ELISA kit from Immunotag^™^ G-Biosciences (Miss The proinflammatory and anti-inflammatory cytokines level y cytokines was checked using Bio-Plex 23 panel) Pro^™^ Cytokine multiplex kit (Bio-Rad, California, United States). Primary and secondary antibodies for TNF-α were procured from Cell Signaling Technology, Danvers, MA. Primers for real-time PCR analysis, designed by NCBI Primer blast and synthesized by Sigma-Aldrich (St. Louis, United States).

### 2.2 Preparation of PL02 formulation

The PL02 formulation consisted of a combination of hydrolyzed collagen and a herbal blend. The herbal blend included standardized extracts of Rosehip (*Rosa canina* L) and Seabuckthorn (*Hippophae rhamnoides*), enriched with folic acid, omega-3 fatty acids, omega-7 fatty acids, omega-9 fatty acids, vitamin C, and alpha-lipoic acid.

For administration, the PL02 formulation was orally administered daily at a dosage of 600 mg/kg body weight, dissolved in MilliQ water. In the herbal mixture, we used a ratio of 60 parts *Hippophae rhamnoides* (Seabuckthorn) extract to 40 parts *Rosa canina* (Rosehip) extract. In the PL02 formulation, we used a ratio of 80 parts hydrolyzed fish collagen type 1 to 20 parts of the herbal mixture.

#### 2.2.1 Collagen standardization for quality control

To enhance the biological activity of the peptide and remove excess salts, purification was performed using the Amersham Pharmacia Fast protein liquid chromatography (GE AKTA Explorer) system. The system was equipped with a Pump P-920 controlled by a UPC-900 monitor and an IV-908 injection valve for sample injection. The XK 16/70 column connected to the controller was packed with sephadex G-10/15 beads that had been swelled in MilliQ water overnight at 4°C.

The collagen peptide was dissolved in MilliQ water, and a stock solution of 500 mg/mL was prepared. The solution was then passed through the column at a flow rate of 2 mL/min. Peak fractionation was set using the UNICORN method wizard, and pump A and B inlets were immersed in sonicated MilliQ water. The eluted material’s absorbance at 214 nm was continuously measured using a Zn Lamp, and fractions were collected using the FRAC-900 fraction collector.

The collected fractions were subsequently lyophilized using an Allied Frost Indian fabricated Lyophilizer under a vacuum pressure of 0.02 mbar. This process facilitated the removal of moisture, resulting in the production of a purified peptide product.

#### 2.2.2 MALDI-TOF mass spectrometry

The purified collagen peptide powder was subjected to further analysis using the Applied Biosystems/MDS SCIEX 4800 MALDI TOF/TOF^™^ Analyzer (California, United States) equipped with a 355 nm pulsed Nd:YAG laser. The measurements, both in positive and negative ion detection, were conducted in the reflector mode with delayed extraction conditions. The extraction voltage was set to 20 kV, and gated matrix suppression was implemented to prevent detector saturation caused by matrix ions with m/z values lower than 200.

For each mass spectrum, one hundred single laser shots were averaged. The laser was randomly moved over the sample to account for analyte/matrix variations and homogenize the measurements. It is important to note that the level of detection (LOD) of MALDI-TOF-MS for a specific compound is primarily influenced by the chemical background noise. Therefore, increasing the number of laser shots does not significantly enhance the spectral quality (20).

#### 2.2.3 UPLC-MS analysis

The formulation analysis was conducted using the ACQUITY UPLC Q TOF MS/MS system (Waters Corp., Milford, MA, United States). The chromatography was performed on an Acquity BEH C18 column (dimensions: 100 mm × 2.1 mm, 1.7 μm) at a temperature of 25°C. The mobile phase consisted of three components: (A) 0.1% formic acid, (B) acetonitrile, and (C) methanol, with a flow rate of 300 μL/min. A sample injection volume of 5 μL was used.

The gradient elution program was optimized as follows: initially, 90% B and 10% C, which was then changed to 80% B and 20% C within 2 min. This was followed by a range of 50–60% B and C for 1–3 min, 30–70% B and C for 3–6 min, 10% B and 90% C for 1 min, and finally rapidly increased to 90% B and 10% C within 7–10 min. The MS analysis was conducted in positive ion mode. Centroid mode data were collected in the mass-to-charge ratio (m/z) range of 100–1,000 Da, with a scan time of 1 s. For data analysis, the Software Respect for Phytochemicals^1^ was utilized.

### 2.3 Experimental design

The experiments were carried out using two rodent models (mice and rats) of both sexes, aged 8–10 weeks in a double blinded manner. Initially, a pilot efficacy study was conducted in mouse models, employing the carrageenan-induced inflammatory model to ascertain the optimal effective dose of PL02. Once the effective dose of PL02 was determined, we proceeded to induce MIA-induced osteoarthritis in mice, as outlined in the Supplementary Figure S5. Subsequently, the identical experiment with the established dose was validated using a rat model.

Wistar rats were randomly divided into four groups (*n* = 10 each) as follows: Sham control group (Vehicle; *n* = 10) received saline instead of MIA injection, disease control group (MIA; *n* = 10) induced with OA and treated with vehicle, positive control group (MIA + Indomethacin [20 mg/kg]; *n* = 10) induced with OA and treated with orally administered Indomethacin, and formulation PL02 treated group (MIA + PL02; *n* = 10). Pain and inflammation were assessed by performing behavioral tests on days 3, 7, 14, 21, and 28. All animal studies were approved by the Institutional Animal Ethics Committee (IAEC#576/21) of the National Institute of Immunology and conducted in accordance with the ARRIVE (Animal Research: Reporting of *in vivo* Experiments) guidelines.

**Figure 1:**
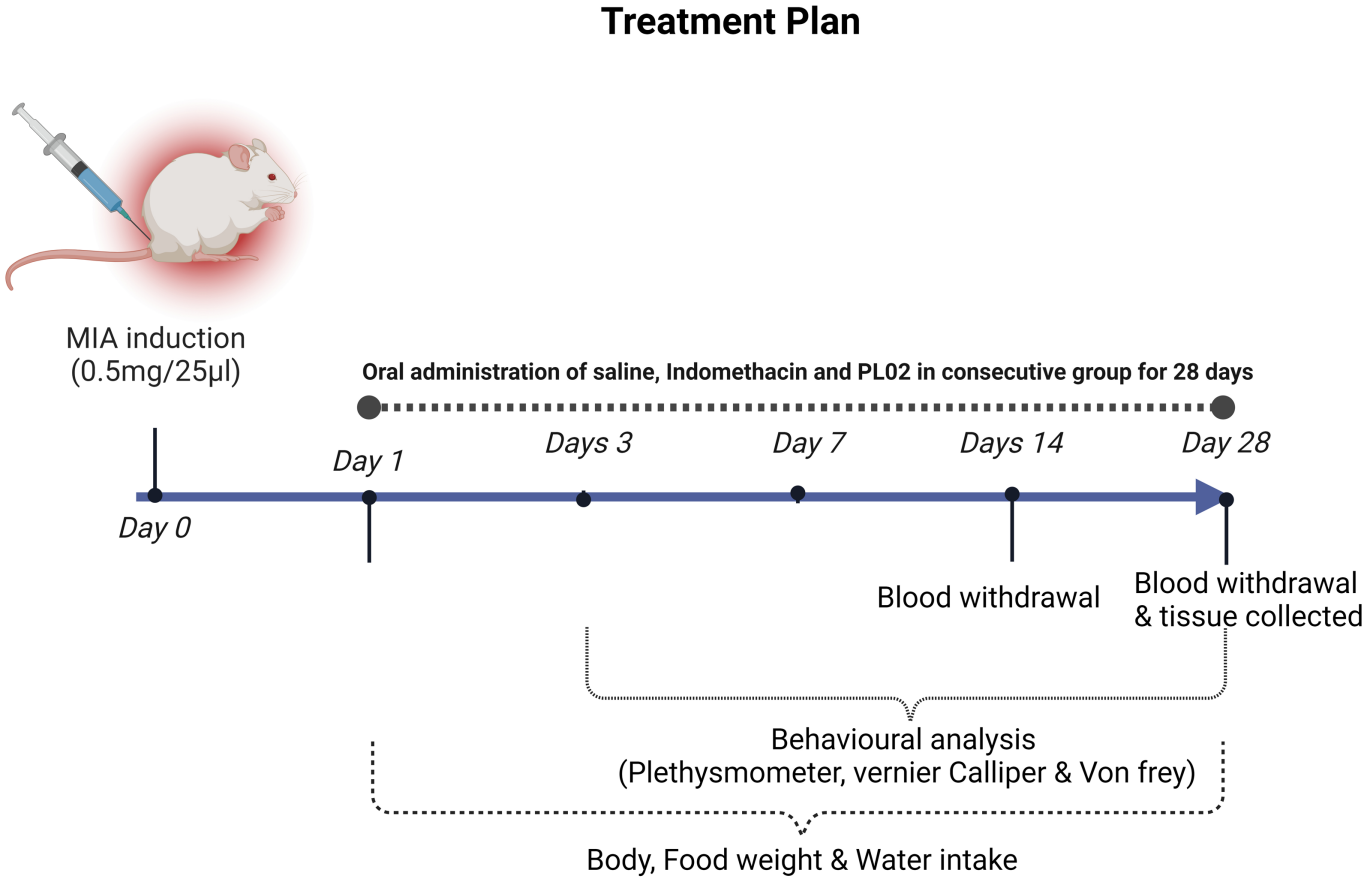
Image illustrated the treatment strategy and analysis plan chart of the study.

The MIA-induced OA rat model was developed following a previously reported procedure (21). To administer MIA (Sigma, St. Louis, United States) intra-articularly, the left knee of the rats was shaved and disinfected with 70% alcohol after anesthesia induction using xylazine (10 mg/kg, intraperitoneal) and ketamine hydrochloride (80 mg/kg, intraperitoneal). After local trichotomy, the leg was flexed, maintaining the knee at a 90° angle, and a 29G needle attached to a 0.3 mL insulin syringe was inserted through the intra-patellar ligament to deliver a single intra-articular injection of MIA (0.5 mg/25 μL in 0.9% sterile saline). The Sham control group received an injection of 25 μL saline only (22) (Figure 1).

Animals were observed on specified days for pain and inflammation in the knee joint and paw. After 4 weeks of treatment, the rats were euthanized using carbon dioxide (CO2) inhalation, following a fill rate of about 30 to 70% of the chamber volume per minute. Blood, vital organs, and left knee joint specimens were collected for further experiments. Blood samples were centrifuged at 12,000 rpm for 10 min, and the serum was separated, aliquoted, and stored at −20°C for subsequent analysis. Synovial tissue and left knee joints were processed for mRNA isolation, lysate preparation, and histological analysis or stored at −80°C for future studies.

#### 2.3.1 Mechanical allodynia

Von Frey is regarded as the gold standard technique used to evaluate the pain sensitivity of the hind paw of the animals with knee joint arthritis. Paw withdrawal threshold (PWT) is measured in response to increasing pressure stimuli applied by von frey hairs to the plantar surface (23). Electronic von Frey Anesthesiometer (IITC Inc. Life Science, California, United States) consists of rigid Supertips^™^ mounted on a sensitive probe. On applying pA, connected LCD Readout will display the weight bared by the paw in grams on applying pressure placed in individual cages with a metal grating floor and allowed a 30 min adaptation before the start of the test. Increasing force was applied to the central plantar region of the hind paw to induce reflecting flexion of the joint. A positive response was defined as a rapid withdrawal of the left hind paw or licking of the paw. The test was repeated three times. The first day of the testing provided a baseline measure of tactile sensitivity. Rats in each group were then tested with von Frey filaments on post-injection 3, 7, 14, 21, and 28.

#### 2.3.2 Measurement of knee edema

The paw diameter was measured after the injection of MIA using electronic Vernier caliper (Insize, India) after MIA injection (24). The difference between inflamed (left knee) and right knee joint was calculated indicating the degree of inflammation and was compared with the control group.

#### 2.3.3 Measurement of paw inflammation using plethysmometer

Inflammation in the paw was measured using a plethysmometer (Paw Volume) Meter (IITC Inc. Life Science, California, United States). The original device was designed to measure paw volume and its changes (swelling) in rodents aiming to test the efficacy of anti-inflammatory agents (25). The left hind paw was dipped in the mercury column attached on a stand and the rise in the mercury level displayed in ml was noted to check for the edema in rat paw.

### 2.4 Biochemical estimation

Blood was withdrawn on day 14 and day 28 through the retro orbital region of rat in the non-anti-coagulant tube. The collected blood samples were centrifuged at 12000 rpm for 15 min at 37°C, and serum was separated from the blood and stored at −20°C.

#### 2.4.1 Toxicity assessment

The measurement of biochemical parameters (Tulip diagnostic Pvt. Ltd. auto analyzer) Serum glutamic oxaloacetic transaminase (SGOT), Serum glutamic pyruvic transaminase (SGPT), Alkaline phosphatase (ALP), Urea, and Creatinine analysis was performed as per mentioned in the manual of Coral clinical system (26).

#### 2.4.2 Quantitative analysis of antioxidant and anti-inflammatory activity in serum

Lipid peroxide, a biomarker for cell oxidative damage, was measured in the rat serum using ELISA kit (Immunotag^™^ G-Biosciences, Missouri, United States). Lipid Peroxidation (LPO) Assay was performed according to the instruction manual provided by the manufacturer. Serum levels of proinflammatory and anti-inflammatory cytokines were analyzed using the Bio-Plex Pro^™^ Cytokine Standard kit (Bio-Rad, California, United States). After thawing the sample, the serum was diluted with Bioplex diluent (1:4). The plate was prepared following the instruction manual provided by the manufacturer and read using Bio-Plex 200 Systems (Bio-Rad, California, United States). The data was analyzed on Bio-Plex Manager (acquisition and Analysis) software at low PMT, RP1 instrument settings (27).

### 2.5 Real time PCR

Total RNA was extracted from bone joint (bone, cartilage and synovium) tissue of all the treatment groups using TRIzol (Invitrogen). 1 μg of total RNA was reverse transcribed using the Verso cDNA synthesis kit (Thermo Fisher Scientific). For real time PCR, data acquisition and analyses were performed using the Light Cycler Real Time PCR system (Roche Diagnostics) and SYBR Green I (Roche Diagnostics, Mannheim, Germany). The relative gene expression levels were normalized against Beta Actin or GAPDH and plotted as fold change to sham control. Primers were designed using the IDT (Integrated DNA technologies) RT primer designing tool. The sense and antisense primers used are given in Supplementary Table S2 (28).

### 2.6 *In situ* assay for biological activity: TNF-α production by SW-982 cells

SW-982 cells (a human synovial cell line SW-982, HTB-93) procured from ATCC were maintained per the provider’s instructions. The anti-inflammatory activity was evaluated as described by Pasi et al. (29). In brief, for *in vitro* assays of the biological activity of PL02, SW-982 cells were plated at a density of 10^5^ cells per well in a 24-well tissue culture plate. After 48 h cells were rinsed thoroughly with PBS, treated with IL-1RA (15 ng/mL) or PL02 (1, 2, 5, 10 mg/mL in PBS, centrifuged at 15000 rpm for 15 min at 4C, clear supernatant passed through 0.2 μm syringe filter, 200 μL/well used for treatment) for 1 h and after that stimulated with 5 ng/mL of human IL-1b (Sigma). After 24 h culture supernatants were analysed for human TNF-a levels using Ultrasensitive ELISA kits from eBioscience as per the manufacturer’s instructions.

### 2.7 Histology

On day 28 after dissections organs such as heart, liver, kidney, brain, and left knee joints are fixed with 10% neutral-buffered formalin for 48 h at room temperature. Decalcification of left knee joints specimens was done with 20% formic acid for 3 days and then embedded in paraffin. Sections of the tissue specimens were acquired from the paraffin blocks at 5 μm thickness, deparaffinized, and rehydrated in the order of xylene, absolute alcohol, and 50% alcohol. The rehydrated sections were stained with hematoxylin and eosin (H&E) to observe morphological changes. Besides H&E stain, the sections of left knee joints used toluidine blue to evaluate proteoglycan and glycosaminoglycans. The severity of articular cartilage injury at the proximal tibiae was scored using the Mankin score (30) with random single blind. The images were visualized and captured using USB 2.0 Camera Viewer (Leadzoptics Microscope, England, United Kingdom).

### 2.8 *Ex vivo* microcomputed tomography (micro-CT) imaging and analysis

Before imaging, fixed left knee joints were further dissected under a dissection microscope to disarticulate them, carefully remove the menisci, and expose the articular cartilage of the tibia and femur. Tibia and femur were subjected to Micro-CT imaging and analysis using μCT (Quantum GXII, Perkin Elmer, United States). Scans were performed using the Aluminium 0.5 + copper 05 filter with the following parameters: time 14 min, Voxel Size 36 μm, tube voltage 90 kV, and tube current 180 mA. Sub reconstruction of the acquired image was also performed to generate Sub-volume vox files with a size of 18 μm. Analyze 14.0 was used to create serial 2D coronal and axial pictures from 3D reconstructed CT scans (Perkin Elmer, United States). We estimated BMD, BV/TV, BV, Tb.Th, and Tb.Sp using analyse14 software by manually selecting the correct region of interest, i.e., lateral and medial subchondral bone in the tibia and femur epiphysis.

### 2.9 Statistical analysis

All experiments were repeated at least three times. Data were presented as mean ± standard error for the indicated numbers of independently performed experiments. Statistical significance was assessed by one-way and two-way analysis of variance coupled with a Bonferroni *t*-test. All statistical analyses were performed using Graph Pad Prism 9.0 for Windows (GraphPad Software, La Jolla, CA, United States).

## 3 Results

### 3.1 PL02 contains collagen peptide and important secondary metabolites

The analysis and validation of collagen peptides were performed using MALDI-TOF. Supplementary Figure S1 demonstrates that the majority of the observed peak masses were <2 kDa, with the highest peak observed at m/z 1879.0494.

**Figure 2:**
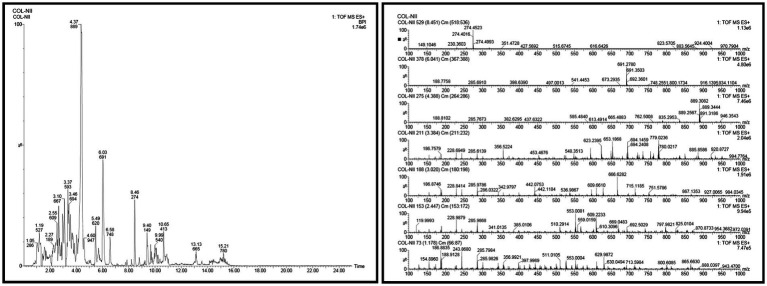
Image illustrated the UPLCMS chromatogram of the PL02 formulation.

In the UPLC Q-TOF MS/MS analysis of the ethanolic extract of *Hippophae rhamnoides* and Rosehip in a chosen fixed ratio, several secondary metabolites were identified (Figure 2). The identification of these compounds was confirmed using the Riken Tandem Mass Spectral Database library. The identified compounds belong to the phenylpropanoid group (including 4 coumaric acid and chlorogenic acid hemihydrate), flavonoid group (chalcone), and alkaloid group (melatonin). The mass spectra, nature of the identified compounds, their structures, and biological activities are summarized in Table 1. These identified compounds have previously been reported for their role in managing pain and inflammation in OA conditions.

**Table 1:** Different compounds of a specific molecular mass detected using UPLC-MS and their uses in osteoarthritic conditions.

S. No.	Compound	Structure	Use	References
1.	(+/−)-alpha-Lipoic acid	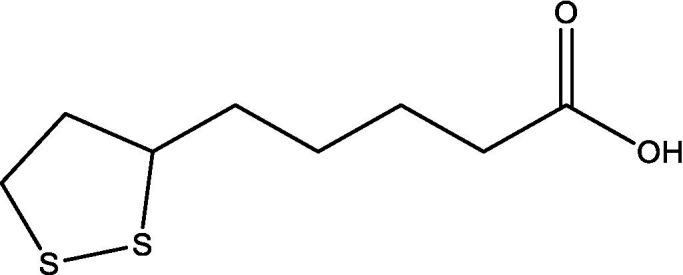	Decrease inflammatory markers such as CRP, IL-6, and TNF-α among patients. It has also been proven as an antioxidant by decreasing the activity of superoxide dismutase and glutathione peroxidase. Available in the market as capsules and tablets as dietary supplements	(31)
2.	Cyanidin chloride	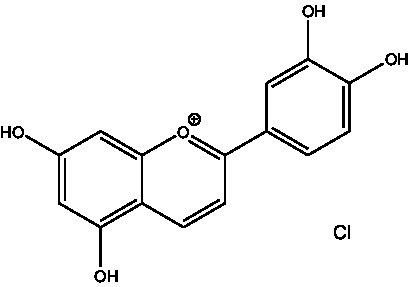	It decreases the IL-6 and interferon (IFN)-*γ* levels and increases the T-reg cell proportion. It possesses antioxidant and radical-scavenging effects	(32)
3.	Kaempferol	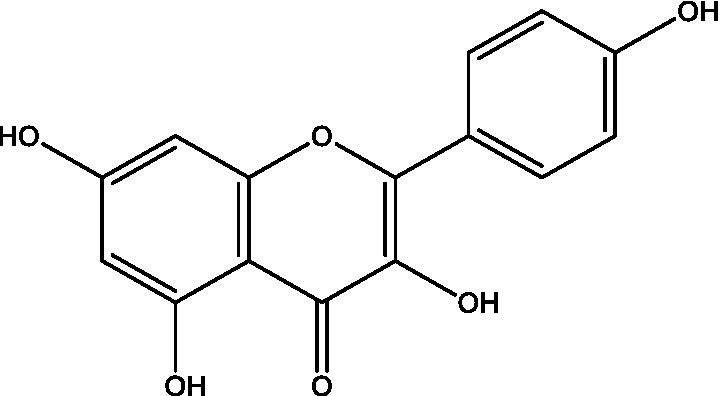	TNF-α, IL-1β, and MDA significantly decreased and a significant increase in SOD level was observed in the ACLT OA animal model. The levels of MMP-13, MMP-3, TNF-α, IL-1β, iNOS were significantly decreased, increased the gene expression levels of collagen IIa1, aggrecan and SOX-9 genes	(33)
4.	Glycyrrhizin	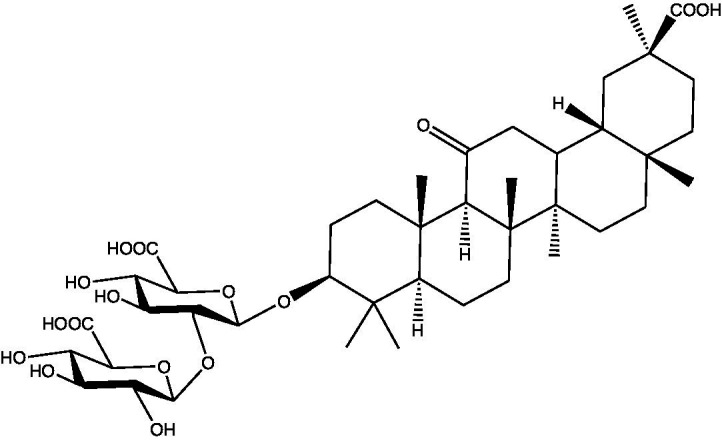	Remarkably suppressed IL-1β-induced level of nitric oxide (NO), prostaglandin E2 (PGE2), TNF-α and IL-6 and the production of cyclooxygenase-2 (COX-2), inducible nitric oxide synthase (iNOs), MMP3, MMP13 and a disintegrin and metalloproteinase with thrombospondin motifs5 (ADAMTS5). Moreover, it significantly inhibited IL-1β-stimulated PI3K/AKT phosphorylation and NF-κB mobilization in human OA chondrocytes	(34)
5.	Resveratrol	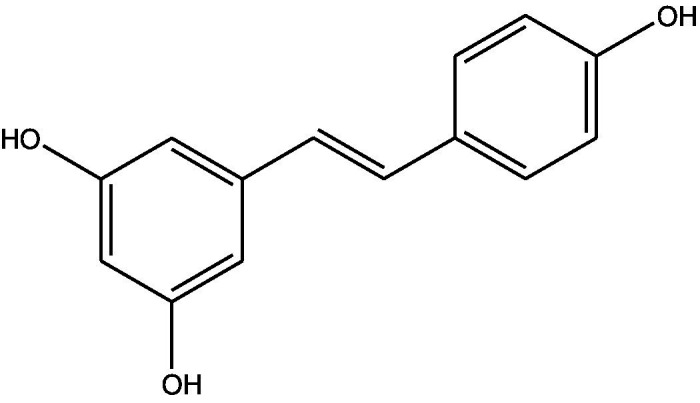	Reduces the expression of inflammatory cytokines in chondrocytes, increased activation of the PI3K/AKT signaling pathway, and regulated expression levels of Bcl-2 and BAX, alleviating the inflammatory response of chondrocytes and a reduction in the apoptosis of chondrocytes. Oral administration at 40 mg (2 caplets) twice a day for one week, then at 20 mg (1 caplet) twice a day, for a total duration of 6 months. Primary knee OA is currently under clinical trial phase 3. Resveratrol can activate SIRT 1 to inhibit OA disease progression. Sirtuin 1 (SIRT 1) is a longevity gene related to many aging-related diseases	(35)
6.	2′,4′-dihydroxy-6′-methoxychalcone	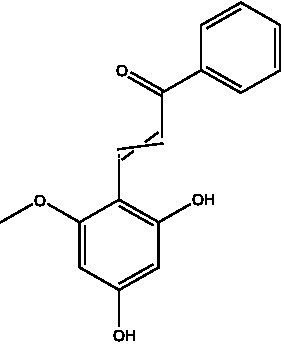	Inhibits the release of pro-inflammatory cytokines such as TNF-α, IL-1β, and IL-6 *in vitro*	(36)
7.	Luteolin	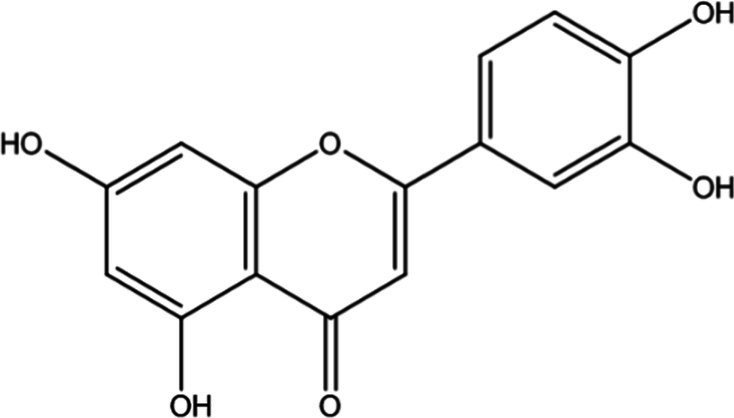	Reduces the IL-1β-induced production of NO, PGE2, TNF-α, MMP-2, MMP-8 and MMP-9 and the expression of COX-2, iNOS, MMP-1, MMP-3 and MMP-13. Luteolin reversed the degradation of collagen II induced by IL-1β. Luteolin treatment prevented cartilage destruction and enhanced collagen II expression in OA rats *in vivo*	(37)
8.	Folic acid	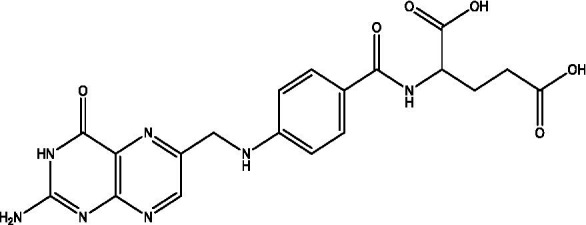	Valuable for cardiovascular disease prevention in RA patients shortly with respect to homocysteine reduction along with blockade of subsequent oxidative stress, lipid peroxidation, and endothelial dysfunction	(38)
9.	Glucocerebrosides	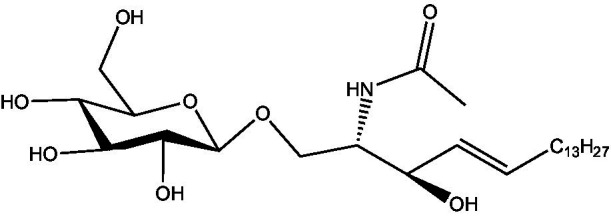	Soya-cerebroside reduces IL-1β-induced MMP-1 production in chondrocytes, without cytotoxic effects, reduced MMP-1 expression via the focal adhesion kinase (FAK), mitogen-activated protein kinase (MEK), extracellular signal-regulated kinase (ERK) and AP-1 signaling pathways, prevents cartilage degradation	(39)
10.	Retinol	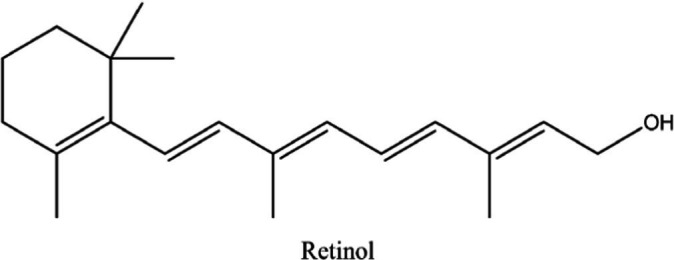	Dietary intervention of vitamin A benefits OA patients 650–750 mg/day (Europe); 700–900 mg/day (United States)	(40)
11.	Syringetin	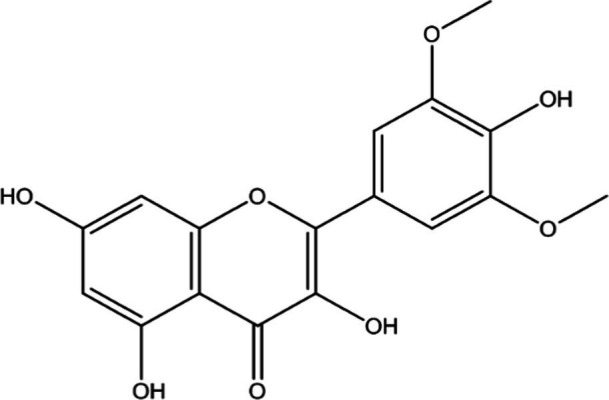	Syringetin stimulates osteoblast differentiation at various stages, from maturation to terminally differentiated osteoblasts. Induction of differentiation by syringetin is associated with increased bone morphogenetic protein-2 (BMP-2) production. Induction of differentiation by syringetin is associated with increased activation of SMAD1/5/8 and extracellular signal-regulated kinase 1/2 (ERK1/2). This will lead to an increase of bone mass	(41)
12.	Diosmin	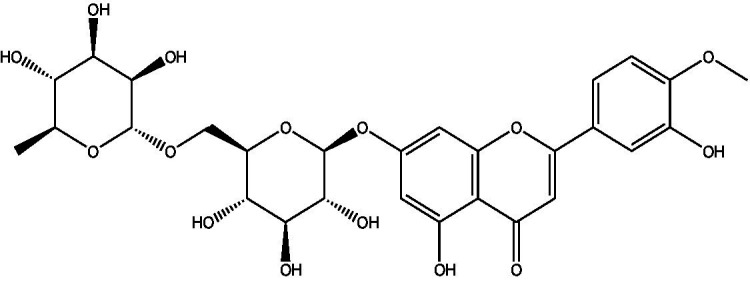	Diosmin down-regulated the mRNA levels of iNOS, COX-2, IL-1β, COL1A1, MMP-3, and MMP-9; up-regulated TIMP-1 and SOX9; and improved COL2A1 in chondrocytes under oxidative stresses. Furthermore, diosmin also regulated glutathione reductase and glutathione peroxidase of H2O2-exposed chondrocytes. Tablets are also recommended for various chronic venous diseases	(42)
13.	L-Carnosine	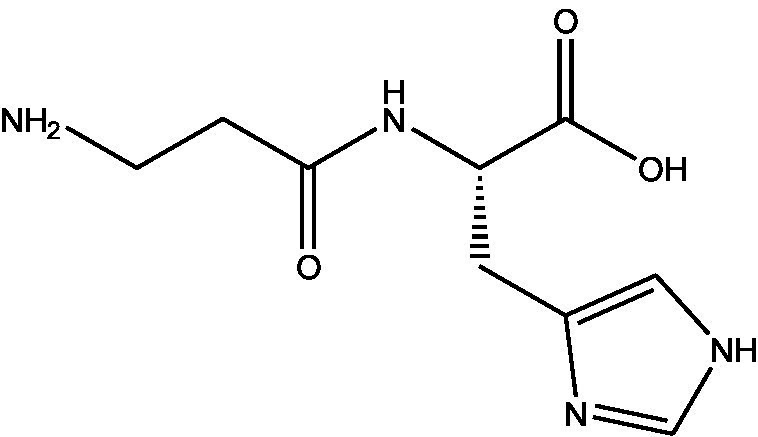	Decreased degree of oxidative damage and reduced levels of proinflammatory cytokines and chemokines and cyclo-oxygenase COX-2 enzyme has been reported. Dietary supplementation of carnosine is advised for human nutrition and health	(43)
14.	Calciferol	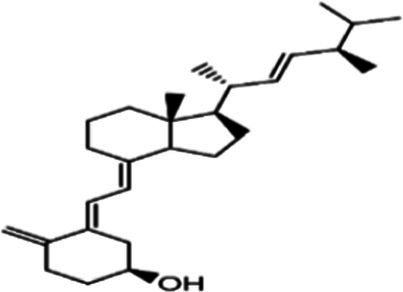	Vitamin D deficiency has been linked to diffuse musculoskeletal pain. It is available in the market as sachet and tablets to treat vitamin-D deficiency	(44)
15.	Hinokitiol	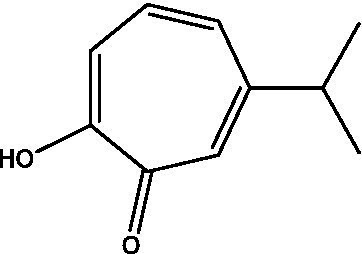	Inhibited IL-1β-stimulated MMP-1,-3 and − 13 expressions and IL-1β-induced activation of intracellular β-catenin proteins	(45)
16.	Trans-Cinnamic acid	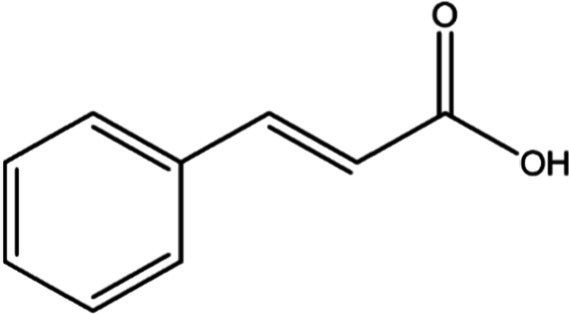	Pretreatment decreased the degradation of IκBα and increased the expression of p-IκBα, indicating that NF-κB inactivation and significantly decreased expression levels of MMP-1, MMP-3, MMP-13, ADAMTS-4, and ADAMTS-5	(46)
17.	Solasodine	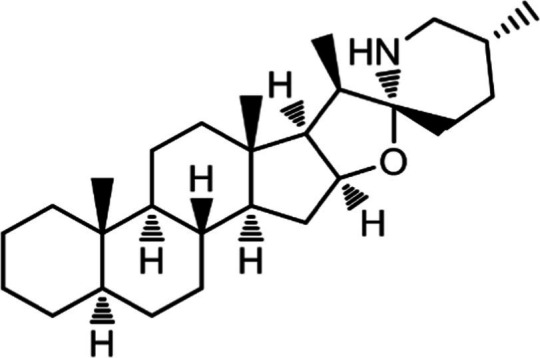	Solasodine exerted statistically significant and dose-dependent anti-inflammatory activity in carrageenan induced rat paw oedema. It also suppressed the volume of exudates, total leucocytes and amount of neutrophil migration into the rat pleural cavity, solasodine exerts anti-inflammatory activity, at least partly through the inhibition of cyclooxygenase and 5-lipoxygenase pathways	(47)
18.	Orientin	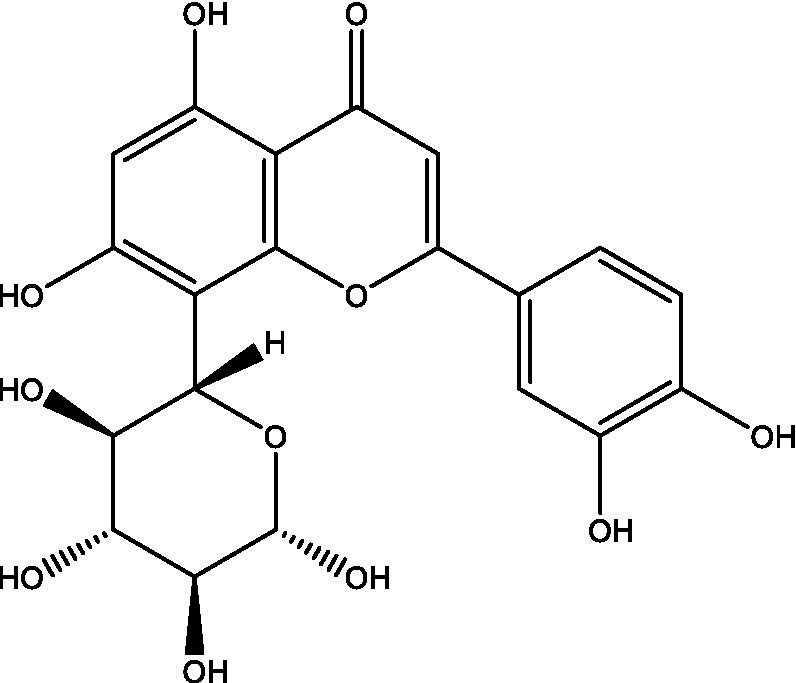	Inhibitory effects were observed on proinflammatory cytokine generation like TNF- α, IL-6, IL-18, and IL-1β, along with PGE2. The expression levels of COX-2 and inducible iNOS were also reduced. Further study demonstrated that such inhibitory effects of Orientin were due to suppression of the NF-kB pathway and nucleotide-binding domain- (NOD-) like receptor protein 3 (NLRP3) inflammasome activation, which may contribute to its anti-inflammatory effects	(48)
19.	Fortunellin	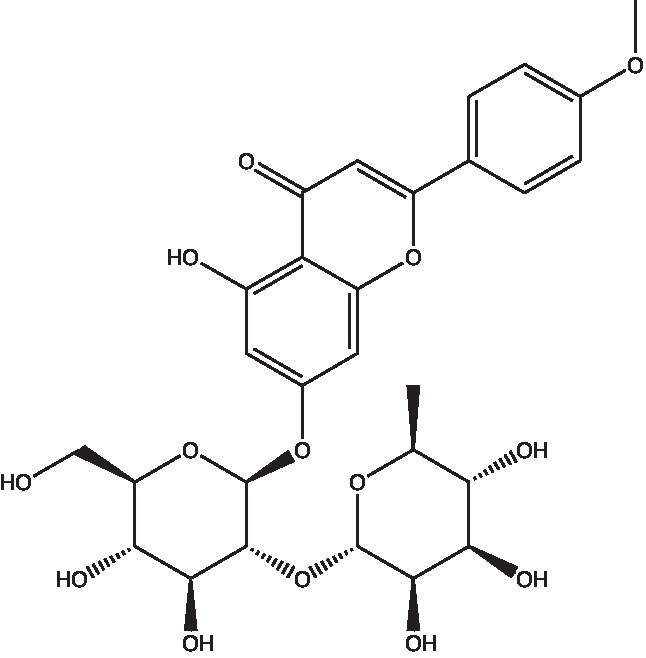	Proven Antioxidant effects	(49)

### 3.2 PL02 exhibited no toxicity in mice and rats after 28 days of treatment

The toxicity study was conducted in both mouse and rat models. To assess acute toxicity, mice and rats were orally administered a dose of 2 g/kg and observed for 24 h. No mortality was observed, and clinical signs such as temperature, changes in skin and eye color, general physique, diarrhea, and sedation were recorded (Supplementary Table S1).

In the mouse study, changes in body weight were observed on days 1, 3, 7, 10, 14, and 28, and biochemical assays were performed on days 14 and 28. The mean body weight was significantly reduced (*p* < 0.001) compared to the sham control and MIA groups (Supplementary Figure S2A), although no physical or behavioral changes were observed. After treatment with PL02 and Indomethacin, the animals exhibited a similar pattern of increased body weight as the normal control. In the biochemical assays on days 14 and 28, the levels of SGOT, SGPT, ALP, Urea, and Creatinine showed significant changes (*p* < 0.001) compared to the sham control and MIA-treated groups, but the values were within the physiological range (Supplementary Figure S2B).

**Figure 3:**
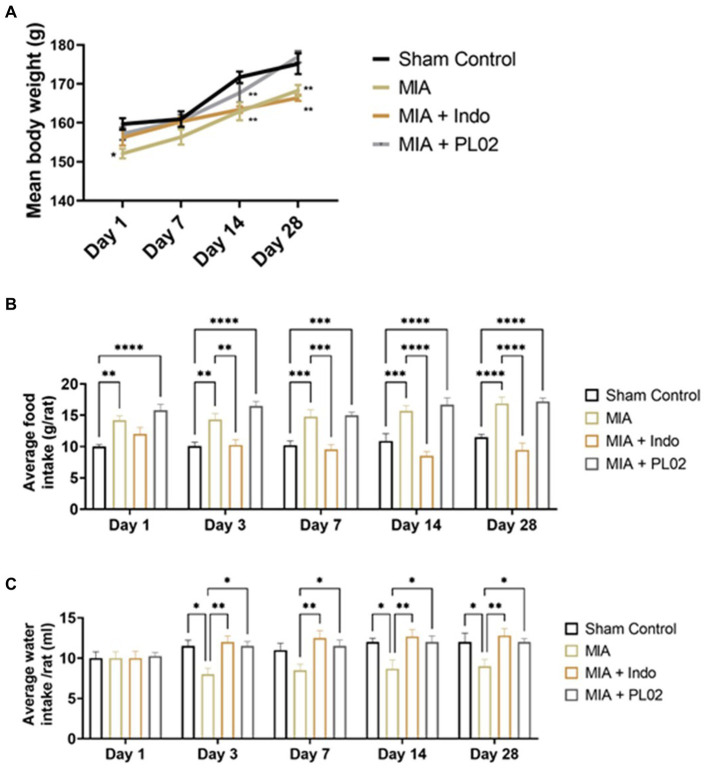
Body weight food and water intake of rats during interventions. **(A)** Average body weight recorded at days 1, 3, 7, 14, and 28 (*n* = 10). **(B)** Average food intake recorded at days 1, 3, 7, 14, and 28 (*n* = 10). **(C)** Average water intake volume was recorded at days 1, 3, 7, 14, and 28 (*n* = 10). Statiscal analysis was performed using two-way ANOVA with Bonferroni *post hoc* test. Data presented as means ± SEM. *p < 0.05, **p < 0.01, and ***p < 0.001.

Subsequently, the toxicity studies were extended to a rat model of OA, including the evaluation of histopathological changes in vital organs. Body weights were recorded on days 1, 7, 14, and 28. A significant reduction in body weight was observed in the MIA-treated group compared to the sham control, while the PL02 treatment improved body weight on day 14 and day 28 (*p* < 0.05, *p* < 0.01, and *p* < 0.001) (Figure 3A). The average food intake showed significant changes (*p* < 0.01, *p* < 0.001, and *p* < 0.001) at each time interval for the MIA and MIA + PL02-treated groups compared to the sham control. However, the MIA + Indo group exhibited significant changes (*p* < 0.01 and *p* < 0.001) on day 14 and day 28 compared to the sham control, MIA, and MIA + PL02 groups (Figure 3B).

The average water intake volume was also measured throughout the study. A significant reduction in water intake was observed in the MIA-treated group (*p* < 0.05 and *p* < 0.01) on day 7, day 14, and day 28 compared to the sham control, MIA + Indo, and MIA + PL02 groups (Figure 3C).

To assess kidney and liver functions, which are indicators of toxicity, biochemical analysis of serum samples was performed. The estimation of SGOT, SGPT, ALP, Urea, and Creatinine on day 14 and day 28 in the serum of rats showed significant changes in ALP level [Supplementary Figure S4A(iii)], Urea level [Supplementary Figure S4A(iv)] on day 14, and creatinine level [Supplementary Figure S4A(v)] on day 14 and day 28 in the MIA group compared to the sham control. However, the values were within the normal range. These observations collectively indicate no toxicity in rats treated with the PL02 formulation.

To further confirm the toxicity of PL02, histological analysis of vital organs was performed on day 28. H&E staining of the heart tissue showed no structural loss of myocytes, and the endothelial cell nucleus appeared normal [Supplementary Figure S4B(i)]. The brain tissue, particularly the hippocampus region, exhibited healthy neurons and glial cells, and the dentate gyrus region showed enriched neurons in the MIA + PL02 treated group compared to the MIA or control groups [Supplementary Figure S4B(ii)]. In the liver tissue, the MIA-treated group showed flaccid hepatocytes, while the MIA + PL02 group did not exhibit any cell death after 28 days of treatment [Supplementary Figure S4B(iii)]. The MIA group displayed an increased size of the glomerulus in the kidney tissue section, while the MIA + PL02 group showed similar patterns of renal corpuscles, tubules, and collecting ducts compared to the control [Supplementary Figure S4B(iv)].

### 3.3 Standardization of MIA-induced animal model of OA

We closely monitored the animals after MIA injection for visible signs of inflammation during the first 24 h. We used various methods to assess different aspects of the condition, including a plethysmometer to measure edema, Von Frey hair to evaluate mechanical allodynia, vernier caliper to measure knee joint thickness, hot plate to measure hyperalgesia, and Rotarod (for mice) to assess motor function at 1, 3, 5, 7, 14, 21, and 28 days.

**Figure 4:**
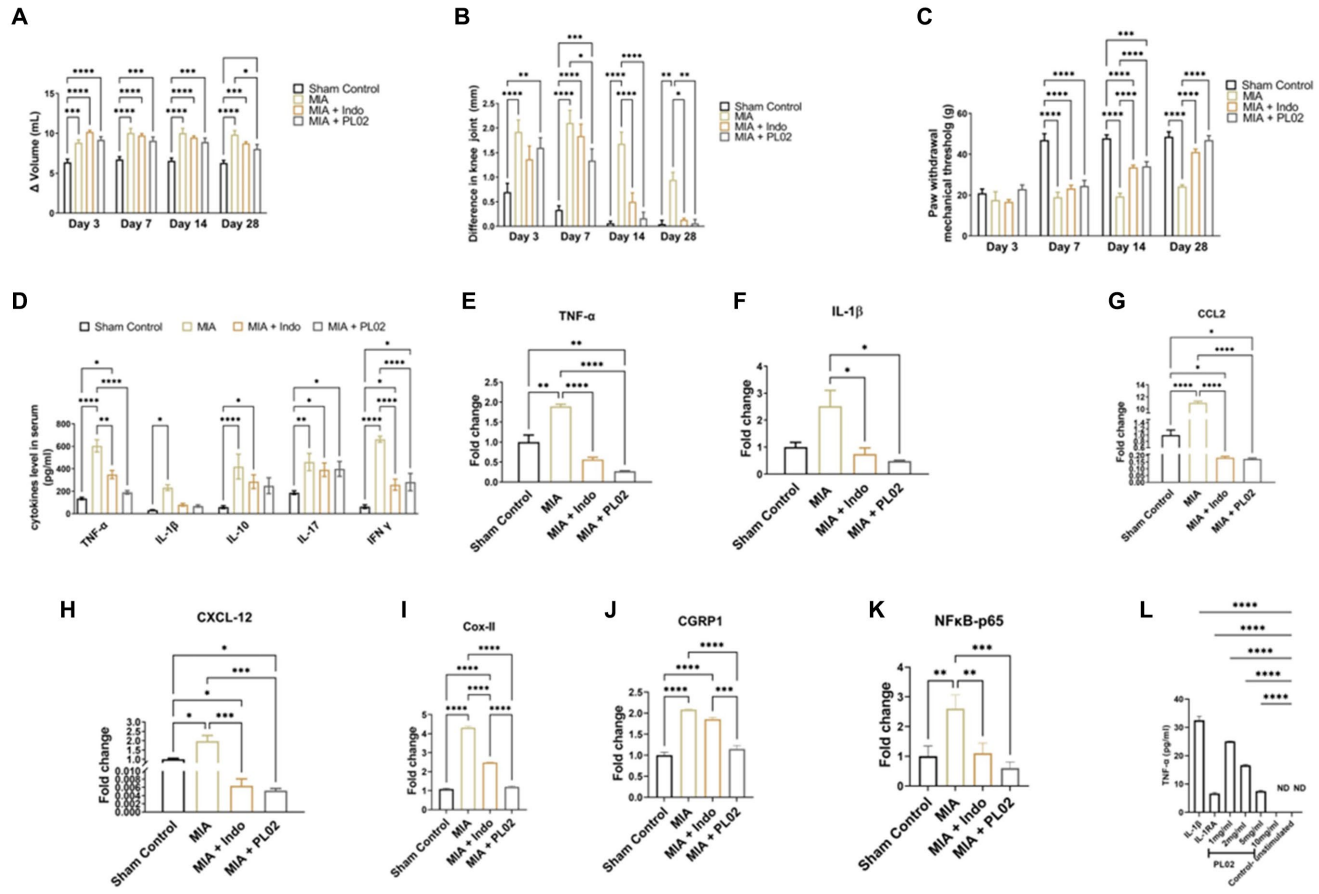
PL02 helps in ameliorated the pain and inflammation **(A)** Plethysmometer used to measure the inflammation of paw edema at days 3, 7, 14, and 28 (*n* = 10). **(B)** Vernier caliper used to the measure the inflammation on knee joint at days 3, 7, 14, and 28 (*n* = 10). **(C)** Von frey was performed for mechanical allodynia to measure the pain at days 3,7, 14, and 28 (*n* = 10). **(D)** Serum cytokines levels measured through ELISA method after 28 days treatment (*n* = 6). **(E–K)** Quantitative real-time PCR analysis of TNF-α, IL-1β, CCL2 CXCL2, Cox II, CGRP1, and NF kB-p65 mRNA levels (*n* = 6). **(L)** *In situ* assay for biological activity: TNF-a production by SW-982 cells (*n* = 3). Statistical analysis was performed using two-way ANOVA and one way ANOVA with Bonferroni *post hoc* test. Data presented as means ± SEM. *p < 0.05, **p < 0.01, and ***p < 0.001.

Visible inflammation was observed 2 h after MIA injection and continued to increase until day seven, with no significant changes observed until day 14. From day 14 to day 28, a decrease in inflammation was observed, although it was not statistically significant. TNF-α and IL-1β levels in the serum were measured at 1, 3, 7, 14, 21, and 28 days, and their levels remained significantly higher throughout the 28 days period compared to the sham control (Figure 4D). Additionally, the NFκB-p65 and IL-1β mRNA levels in the joint tissue were assessed at day 7, 14, 21, and 28. No significant change was observed in the CTX-II level. However, the mRNA levels of NFκB-p65 and IL-1β showed a significant increase up to the 28th day (NFκB-p65: 2.7 ± 0.82-fold change; IL-1β: 10 ± 1.79-fold change) compared to the sham control. These findings collectively confirmed the successful development of the OA model.

For more detailed studies, we evaluated pain, inflammation symptoms, and behavior at 3, 7, 14, 21, and 28 days. Blood samples were collected, and the animals were euthanized on day 28 for molecular and histological analysis. Joint sections from the MIA-induced animals exhibited OA-like features, including surface discontinuity, loss of proteoglycans, disorientation, apoptosis of chondrocytes, and subchondral bone sclerosis (21).

### 3.4 PL02 ameliorated pain and inflammation in MIA-induced OA

In mice, we assessed hyperalgesia using the hot plate method to analyze pain sensation. The changes in latency time were found to be non-significant on day 1 and day 3 in the MIA + PL02 treated groups compared to the MIA group. However, after 10 days of PL02 treatment, a significant increase in latency time was observed from *p* < 0.05 to *p* < 0.0001 on day 28 (Supplementary Figure S3A). The latency time increased from (22.56 ± 6.76) to (50.11 ± 16.54) in the MIA + PL02 group, in comparison to the sham control group (11.25 ± 4.79) to (25.14 ± 12.56) and the MIA group (10.25 ± 8.23) to (10.59 ± 16.58). However, the changes in latency time in the MIA + PL02 group were less significant compared to the MIA + Indo group, ranging from *p* < 0.05 to *p* < 0.01.

To assess motor neuron function, we used the Rotarod test. The latency to fall time significantly increased after 14 days of treatment in the MIA + PL02 group, from (20.75 ± 12.34) to (298.50 ± 73.44), compared to the sham control group (10.75 ± 6.24) to (123 ± 70.03) and the MIA group (8.80 ± 7.26) to (87.11 ± 30.12). These changes showed even more significant improvements after 28 days of treatment (*p* < 0.0001) (Supplementary Figure S3B).

We measured inflammation in the knee area using a Vernier caliper on both knees and noted the difference. On day 1 and day 3 post MIA injection, all groups showed a significant difference in knee width, confirming the development of an osteoarthritis model. However, a significant decrease in the difference between the two knee joint widths was observed from day 10 onwards in the PL02 treated group compared to the MIA and Indomethacin treated groups (Supplementary Figure S3C).

Mechanical hypersensitivity was measured using the Von Frey method. The MIA + PL02 group showed a more significant (*p* < 0.0001) improvement after 10 days of treatment, increasing from (3.65 ± 0.44) to (5.09 ± 1.46) on day 28, compared to the MIA treated group (Supplementary Figure S3D). These results from the initial pilot study on a mice model of OA confirmed the superior efficacy of PL02 over indomethacin.

The anti-inflammatory property of the PL02 formulation in the rat model was assessed using the Plethysmometer. By immersing the paw in the mercury column, the change in volume (Δ) of the left paw indicated elevated inflammation in rats administered with MIA. However, the group administered with the PL02 formulation showed anti-inflammatory activity, significantly reducing inflammation on day 28 from (9.19 ± 0.39) to (8.07 ± 0.56) in the rat’s paws, compared to the MIA group (Figure 4A).

In Figure 4B, the diameter of the knee joint significantly increased post-MIA injection compared to the sham control group. The left knee joint administered with MIA exhibited elevated inflammation, while the right knee was used as a control. The difference in width between the right and left knee joint was measured to evaluate the anti-inflammatory property of the PL02 formulation. A gradual decrease in inflammation in the knee joint was observed in the MIA + PL02 group from (1.6 ± 0.20) to (0.06 ± 0.07) and the MIA + Indo group from (1.36 ± 0.20) to (0.13 ± 0.03), compared to the MIA group from (1.9 ± 0.24) to (0.94 ± 0.15) after 28 days of treatment with the PL02 formulation.

To assess mechanical allodynia, which is a hallmark of the MIA-induced OA model, the von Frey hairs test was used. The paw withdrawal threshold, or the weight bared by the rat paw in grams displayed on a digital meter, was noted. The PL02 formulation exhibited analgesic effects starting from day three, with the paw withdrawal threshold in the MIA + PL02 group being higher than in the MIA + Indo treated group. A significant increase (*p* < 0.0001) in the paw withdrawal threshold was observed from the 14th day onwards in the MIA + PL02 treated group and the MIA + Indo treated group, compared to the MIA group. The pain significantly decreased further with daily administration of the formulation for 28 days (*p* < 0.0001) (Figure 4C).

After 28 days of treatment, blood was withdrawn from the rats and used for cytokine assay using a multiplex analyzer (Figure 4D). The level of TNF-α was significantly (*p* < 0.0001) decreased in the MIA + PL02 group compared to the MIA group. The level of IL-1β also significantly reduced upon PL02 treatment compared to the MIA group. Similar patterns were observed for IL-10 and IL-17 levels, with the PL02-treated group showing lower levels than the MIA group, although the changes were non-significant after analysis. However, the levels of IFN-γ were significantly (*p* < 0.0001) reduced in the MIA + PL02 group compared to the MIA group.

To further validate the serum cytokine levels, we checked the mRNA expression in left knee joint tissue using real-time PCR analysis of pro-inflammatory cytokines, including IL-1β, TNF-α, and NFκB-p65. The knee joint with a complete synovial capsule was used to evaluate the effect of PL02 on the joint. The mRNA expression of pro-inflammatory mediators IL-1β (Figure 4F), NFκB-p65 (Figure 4K), and TNF-α (Figure 4E) significantly decreased in the PL02-treated rats compared with the MIA group (*p* < 0.05) and (*p* < 0.0001), respectively. Chemokines such as CCL-2 (Figure 4G) and CXCL-12 (Figure 4H) also showed significantly declined levels (*p* < 0.0001 and *p* < 0.001) after daily administration of the formulation for 28 days in the PL02-treated group, compared to the MIA group. Additionally, to understand the mechanism, a synovial cell line was used to study IL-1β-dependent TNF-α production. Treatment with PL02 showed a dose-dependent decrease in TNF-α production, and at 10 mg/mL, it inhibited IL-1β-induced TNF-α production (Figure 4L). This indicates that PL02 inhibited the inflammatory cascade at an early stage, reducing pro-inflammatory cytokine production and associated pathology.

It is well established that these pro-inflammatory cytokines enhance the expression of COX-2 and CGRP-1, genes directly involved in pain perception. Therefore, we checked the mRNA expression of these genes in joint tissue. A significant increase (*p* < 0.001) in COX-II and CGRP-1 levels was observed in the MIA group compared to the sham control group. PL02 treatment for 28 days significantly reduced their expression (*p* < 0.001) compared to the MIA and MIA + Indo groups (Figures 4I,J). The expression pattern of COX-2 and CGRP-1 correlated well with the pain behavior data (Figures 4A–C).

### 3.5 PL02 reduced the oxidative stress and inhibited the cartilage destruction

**Figure 5:**
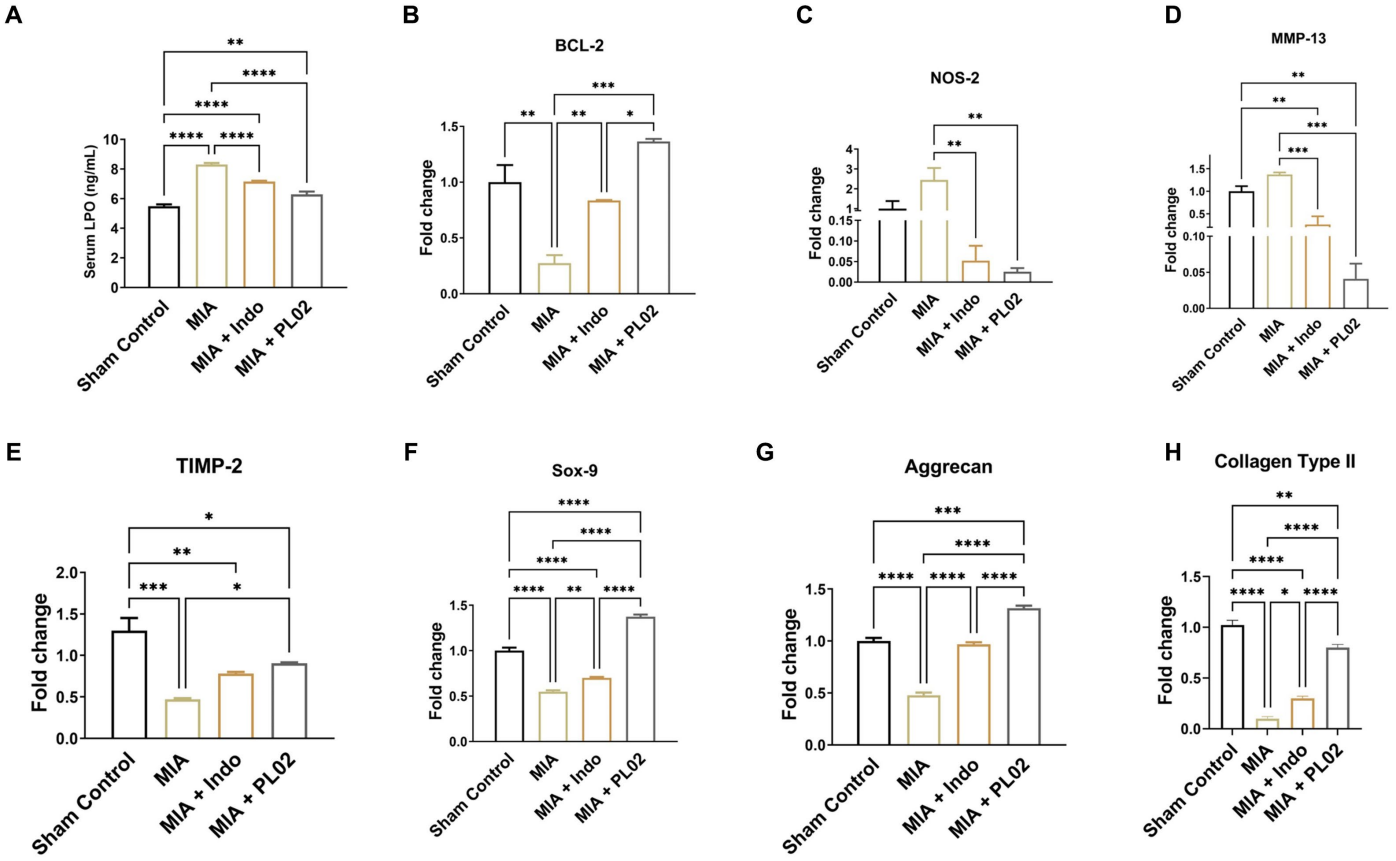
PL02 inhibit cartilage damage by reducing oxidative stress chondrocyte apoptosis and induced chondrogenesis **(A)** Serum level of LPO measured after 28 days treatment (*n* = 6). **(B–H)** Quantitative real-time PCR analysis of BCL-2, NOS-2, SOX-9, MMP-13, TIMP-2, Aggrecan, and Colla-gen Type II mRNA levels (*n* = 6). Statistical analysis was performed using one-way ANOVA with Bonferroni *post hoc* test. Data presented as means ± SEM. *p < 0.05, **p < 0.01, and ***p < 0.001.

The effect of PL02 on oxidative stress was assessed using the biomarker LPO. Serum samples collected from the different treatment groups of animals on the 28th day post-treatment were used to determine LPO levels (Figure 5A). The MIA group exhibited a significant increase in serum LPO levels (8.302 ng/mL) compared to the sham control group (5.494 ng/mL). However, PL02 treatment markedly reduced serum LPO levels to 6.091 ng/mL, indicating its antioxidant properties. In real-time PCR analysis of whole joint tissue, the PL02 group showed a significantly decreased level (*p* < 0.01) of NOS-2 (Figure 5C) compared to the MIA group. The significantly high level (*p* < 0.0001) of BCL-2 (Figure 5B) confirmed the anti-apoptotic property of PL02 compared to MIA, further validating its antioxidant and anti-apoptotic properties.

Next, we examined the level of the protease MMP-13, which attacks the cartilage matrix and leads to its destruction. MMP-13 expression increased following MIA injection compared to the control group. However, a significant downregulation in the level of MMP-13 was observed in PL02-treated animals compared to MIA (Figure 5D). The reverse pattern was observed for TIMP-2 (Figure 5E), an inhibitor of the MMPs family involved in diseases such as arthritis and metastasis. TIMP-2 expression significantly decreased (*p* < 0.001) after MIA injection but increased significantly (*p* < 0.01) upon PL02 treatment. We also assessed the expression level of collagen-II, the primary collagen, and aggrecan, a major proteoglycan in articular cartilage essential for its proper functioning and hydration maintenance. A significant decrease in aggrecan level was observed following MIA injection, with no significant improvement in the MIA + Indo treated animals.

Interestingly, 28 days of PL02 treatment showed a significant increase (*p* < 0.0001) in aggrecan expression (Figure 5G) compared to MIA. Similarly, a considerable decrease in the fold of the Col-II gene was observed in the MIA group compared to the control group. However, the PL02 treatment group exhibited significantly increased expression of Coll-II compared to the MIA group. No significant change was observed in the Indomethacin-treated group compared to MIA (Figure 5H). The expression of COL-II and aggrecan in the PL02-treated group was comparable to the control group, indicating a restoration of healthy chondrocytes and cartilage.

Subsequently, we assessed the mRNA expression level of SOX-9, a master regulator of chondrogenesis. The high level of SOX-9 ensures the proliferation of chondrocytes and is essential for differentiating precursor cells into chondrocytes. The SOX-9 level significantly decreased following MIA injection compared to the control group, with no improvement observed in SOX-9 expression with indomethacin treatment. However, the 28 days treatment with PL02 in rats showed a significantly high level of SOX-9 (Figure 5F) compared to MIA. The increased SOX-9 expression correlates with increased aggrecan and COL-II expression, indicating the chondroprotective and regenerative properties of PL02.

### 3.6 PL02 protected the articular cartilage degeneration

**Figure 6:**
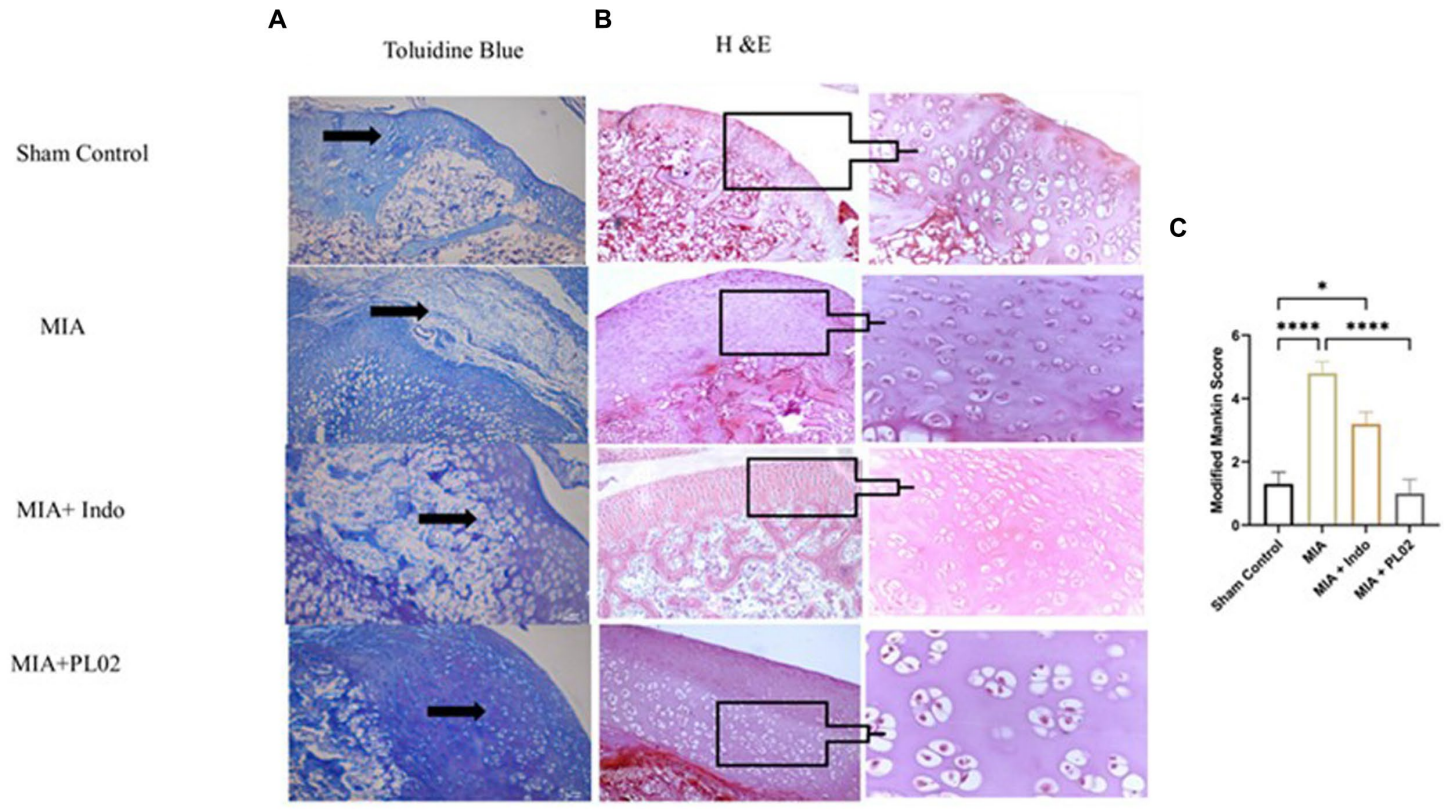
Histological analysis of joint tissue **(A)** Toluidine blue. **(B)** H&E staining of cartilage section was performed to assess the proteoglycan and glycosamnioglycans. **(C)** Modified Mankin score. Scale bar 100 μm.

Histological analysis of toluidine blue (Figure 6A) and hematoxylin & eosin (Figure 6B) staining of knee joint sections was performed after euthanizing the rats on day 28 following intra-articular injection of saline or MIA. In the sham control group, where saline was administered into the intra-articular region of the left knee joint, the articular cartilage surface appeared smooth, and the chondrocytes were arranged in an orderly manner in the superficial, mid, and deep zones of the cartilage. No proliferative changes or distortion of chondrons were observed. However, in the MIA group, after the injection of MIA, superficial fibrillation and loss of the cartilage surface were observed. Chondrosenescence occurred due to MIA injection, leading to fragmentation of chondrocytes within the chondron in the deep zone.

In contrast, significant distortion of the complete chondron was observed in the superficial and mid-zones of the cartilage. Loss of superficial matrix parallel to the surface and the formation of fissures were evident in the H&E-stained distal epiphyseal cartilage of the tibia in the left knee joint of rats. Damage to the bone due to the presence of a subchondral bone cyst was also observed. In the Indomethacin-treated group, cartilage destruction was limited, and only a few chondron deformations were observed. However, daily treatment with the PL02 formulation for 28 days resulted in improved macroscopic changes in the articular cartilage compared to MIA. The cartilage surface appeared smooth, with no irregularities, and the chondrocytes were arranged in parallel zones within well-structured lacunae, confirming the cartilage-protective property of PL02.

Next, we evaluated the levels of proteoglycan and glycosaminoglycan in the joint tissue sections using toluidine blue staining. The staining revealed a significant decrease in proteoglycan and glycosaminoglycan content in the MIA group. However, treatment with MIA + PL02 protected the cartilage from damage, resulting in a significantly increased level of proteoglycan compared to the MIA group (Figure 6A). Interestingly, the improvement in articular cartilage and proteoglycan levels upon PL02 treatment surpassed even those of the sham control group, indicating that PL02 not only prevents chondrocyte apoptosis but also induces regeneration. Our histological observations aligned with the gene expression profile and provided further evidence of the chondroprotective and regenerative nature of PL02.

### 3.7 Subchondral bone analysis

After conducting histopathological analysis, we examined the subchondral bone microarchitecture using micro-CT. All rats treated with MIA exhibited pathological changes in the subchondral bone of the injected left knee. In contrast, the contralateral control knee showed no signs of osteoarthritis (OA)-like changes in the microarchitecture of the tibial subchondral bone. However, we did not observe significant changes in the microarchitecture of the distal/proximal femur bone in the joint following MIA injection. Therefore, we focused on analyzing the tibial subchondral bone in detail.

**Figure 7:**
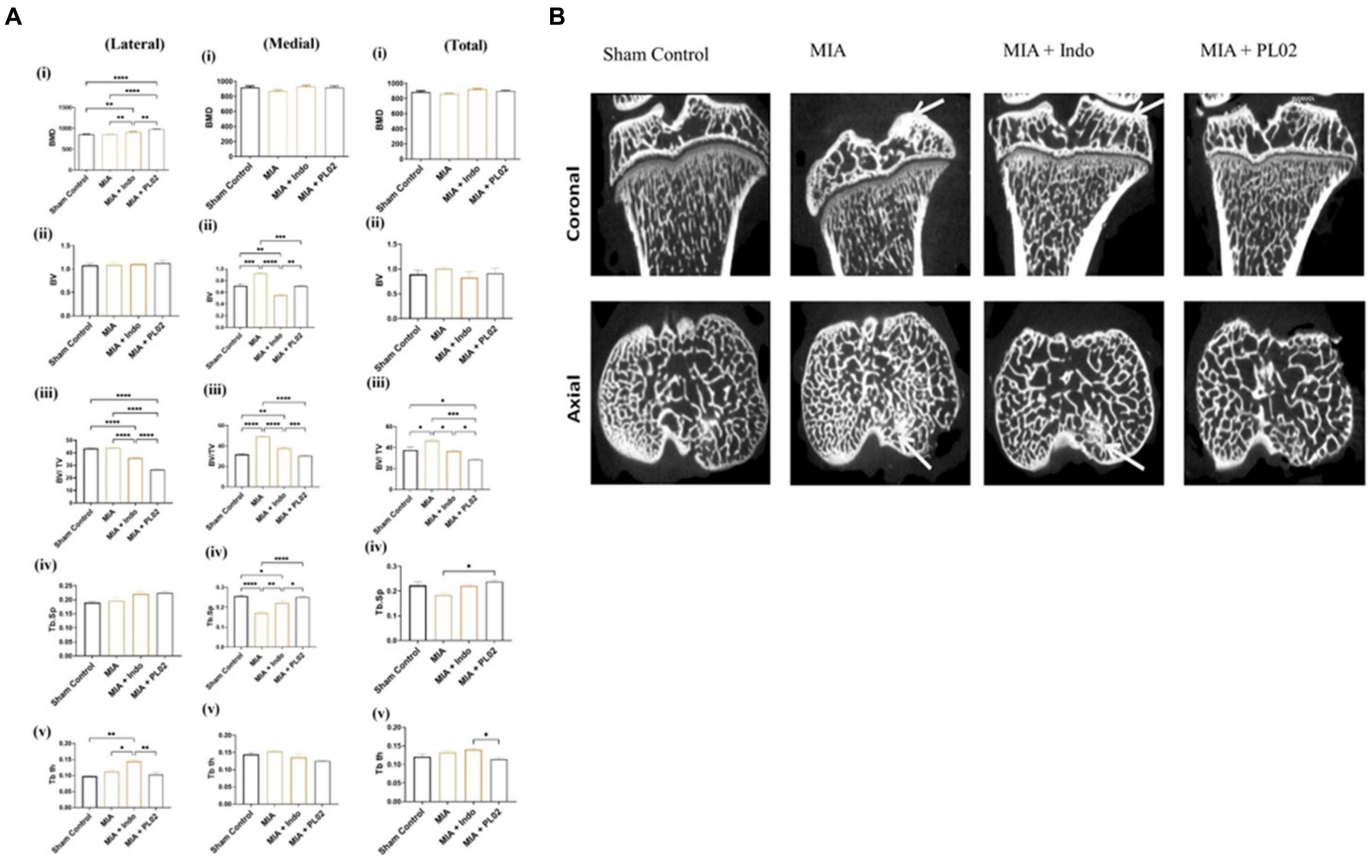
Microarchitectural analysis of subchondral bone **(A)** Bar plot showing morphometric parameters of subchondral trabecular bone de-termined by micro-CT in the control tibiae and in the MIA-injected, MIA injection with treatment, tibiae. There was a statistically significant increase over time in BV, BV/TV, Tb.Th, and Tb.N, and a decrease in Tb.Sp, in both the control and MIA-injected tibiae. Error bars = SD. BV, bone volume; BV/TV, bone volume fraction; CT, computed tomography; MIA, monosodium iodoacetate; TB. N, trabecular number; Tb.Sp, trabecular separation; Tb.Th, trabecular thickness. *p* < 0.05, **p < 0.01, and ***p < 0.001, between control tibiae and MIA-injected tibiae. **(B)** Coronal and axial micro-CT images of control and different treated animal tibia at 4 weeks post treatment, The MIA-injected tibia showed altered subchondral bone architecture, with sclerosis on the medial tibial compartment (indicated by arrow), whereas the control tibia and MIA + PL02 treatment showed no sclerosis at 4 weeks. Micro-CT, micro-computed tomography; MIA, monosodium iodoacetate.

Figure 7A presents a comparison of the tibial subchondral trabecular bone microarchitectural parameters between the control knee, the MIA-injected left knee, the MIA + PL02-treated knee, and the MIA + Indo-treated knee after completing 28 days of treatment. We observed more significant changes in the medial compartment of the tibia compared to the lateral compartment, so we considered the medial and total changes for interpretation. The MIA-injected tibia showed a substantial increase in bone volume (BV) and bone volume fraction (BV/TV) compared to the control tibia, in both the medial and total compartments (15 and 12% increase, respectively; *p* < 0.05 for both parameters). However, no such increase in bone volume fraction was observed in animals treated with PL02, and it was comparable to the sham control. The trabecular parameter Tb-Th slightly increased after MIA treatment, but the difference was insignificant. On the other hand, there was a significant decrease in trabecular spacing (Tb.Sp) in the MIA-treated group compared to the sham control, which was prevented in the PL02-treated group.

The MIA-injected left knee exhibited a significant increase in subchondral plate thickness (in both the medial and total compartments) compared to the control left knee. The calculated values for subchondral plate thickness were 140 ± 25 μm and 120 ± 9 μm for the MIA-injected and sham control knees, respectively. In the PL02-treated tibia, a subchondral plate thickness of 115 ± 15 μm was observed, similar to the sham control. Additionally, we observed increased porosity of the tibial subchondral plate in the MIA-injected left knee compared to the sham control and PL02-treated left knee. The %d MIA-Control was 48, while the %d MIA + PL02-Control was 20 (*p* < 0.001).

Figure 7B displays the lateral and coronal views of the tibia. As depicted in the micro-CT image, increased sclerosis was observed in the MIA-treated tibia compared to the control, and this sclerosis was reduced with indomethacin treatment. Interestingly, no sclerosis was observed in the knees of animals treated with PL02. Overall, the analysis of the tibial subchondral bone indicated that MIA injection induced subchondral pathology similar to that reported in humans, which was prevented by PL02 treatment.

## 4 Discussion

Over the past decade, scientific research in drug discovery has shifted towards a more precise and consistent approach, favoring multitarget strategies for the treatment of chronic degenerative and metabolic diseases. Therapies targeting a single pathway often provide only temporary relief and may have moderate to severe long-term side effects. Osteoarthritis (OA) is a prime example of a chronic medical condition that requires effective treatment. The impact of the ongoing COVID-19 pandemic has further exacerbated the burden of OA. Despite extensive research efforts worldwide, no therapy has been developed that can effectively halt, delay, or reverse OA progression or provide long-lasting symptomatic relief. Consequently, surgical interventions like joint replacement are often pursued by patients seeking improved pain management and quality of life. There is an urgent need to develop a novel, safe, and multitargeted therapy that can address OA symptoms in the long term and prevent or reverse joint tissue loss.

To tackle this challenge, we have utilized our knowledge and expertise in traditional medicine and bone joint physiology to develop a multitargeted formulation called PL02 (patented trademark). The botanical ingredients used in this formulation are already known for their individual health benefits and are widely sold as nutraceuticals worldwide (7, 26, 50). However, their potential as a front-line therapy for OA has not been explored until now. The unique composition of PL02, along with a standardized extraction process conducted at specific temperatures, ensures the maximum presence of flavonoid-rich secondary metabolites. These metabolites, such as alpha-lipoic acid, kaempferol, resveratrol, carnosine, orientin, and others (refer to Supplementary Table S1), have been identified through UPLCMS analysis. The standardized combination of these ingredients has demonstrated a superior synergistic effect in animal models of OA, supporting its potential efficacy.

Our study aimed to establish a non-traumatic animal model that closely resembles the tissue-level pathophysiology of human osteoarthritis (OA). This model would enable us to investigate the effects of disease-modifying therapies on both cartilage and subchondral bone. To achieve this, we utilized a low-dose monosodium iodoacetate (MIA)-induced rat and mouse model of OA, which effectively replicates the articular cartilage and subchondral bone pathology observed in human OA (51). Compared to surgical models of OA, the MIA-induced model is less invasive, more robust, and avoids issues related to symptom variation and infection. Even though MIA induced model has some limitations, it is most widely used pharmacological model for osteoarthritis.

After optimizing the dosage, we conducted acute and subacute (28 days) toxicity studies of PL02 and found no evidence of any toxic effects based on serological and histopathological evaluations. Subsequently, we assessed the efficacy of PL02 in addressing various OA symptoms, including inflammation and pain. Within 1 week of treatment, PL02 demonstrated significant pain relief and reduction of inflammation, surpassing the effects of indomethacin. The superior anti-inflammatory and analgesic activity of PL02 can be attributed to its polyphenols and other secondary metabolites. Notably, alpha-lipoic acid in the formulation played a significant role in controlling inflammation by reducing serum levels of IL-1β, IL-6, TNF-α, IL-17, and IL-23 (31). Additionally, lipoic acid supplementation has been shown to prevent inflammatory bone loss by inhibiting COX-2 activity and PGE2 production (52). Furthermore, kaempferol, another component of PL02, alleviates inflammatory responses by inhibiting iNOS and Cox-2 expression and attenuating NF-kB activation (53).

To evaluate the anti-inflammatory properties of PL02, we measured the knee joint width as an indicator of edema, which is a manifestation of inflammation. Our data from mice and rats showed a significant reduction in knee joint difference (edema) after 14 days of oral PL02 treatment. This finding suggests that PL02 effectively prevented inflammation-associated cell infiltration and fluid accumulation in the knee joint. Furthermore, we assessed thermal and mechanical hypersensitivity as additional indicators of inflammation. The PL02-treated group exhibited a higher threshold of mechanical hypersensitivity, as determined using the Von Frey system, compared to the MIA and MIA + Indo groups. The observed decrease in edema and increase in mechanical threshold provide further evidence of the superior anti-inflammatory and analgesic properties of PL02 compared to Indomethacin.

To gain insights at the molecular level and support our behavioral findings, we examined the levels of pro-inflammatory cytokines in both serum (TNFα, IL-1β, IL-10, IL-17, and IFN-γ) and mRNA expression in joint tissue (TNFα, IL-1β, CCL2, CGRP1, and CXCL-12). The data obtained confirmed the symptom profiles and demonstrated that PL02 significantly ameliorated inflammation and pain by modulating cytokine levels in the serum and joint tissue, surpassing the effects of the MIA or MIA + Indo group. This enhanced anti-inflammatory effect is likely attributed to the synergistic actions of several secondary metabolites present in PL02, as previously described.

Based on the remarkable efficacy of PL02 in alleviating MIA-induced OA symptoms, we delved into understanding the underlying molecular mechanisms. Among the various cytokines, IL-1 has been identified as a potent inducer of cartilage and bone damage, particularly when compared to TNF-α. IL-1 and TNF-α play key roles in inducing matrix-degrading enzymes that lead to rapid and extensive cartilage damage. Therefore, the inhibition of IL-1-induced inflammatory cascades by PL02 has a more pronounced effect on cartilage and bone damage, aligning with the therapeutic goals in OA.

The oxidative stress theory provides an explanation for the key pathogenic events in OA, which involve the degradation of extracellular matrix (ECM) and apoptosis of chondrocytes. Inflammation triggers the generation of reactive oxygen species (ROS) through the activation of multiple signaling pathways. ROS functions both as a signaling molecule and a mediator of inflammation and senescence (52). By acting as second messengers, ROS activate signaling pathways that promote the activation of MMPs, induce cell death, and degrade the matrix, ultimately leading to cartilage degradation. Furthermore, ROS inhibit cell migration, impair the bioactivity of growth factors, and disrupt matrix synthesis, exacerbating cartilage damage (54, 55).

Compounds possessing antioxidant properties have demonstrated the ability to neutralize ROS and interrupt the chain of ROS-mediated damage to cartilage and the synovial capsule (56). In our study, we quantified lipid peroxidation (LPO), a product of ROS-mediated lipid oxidation, and measured the mRNA expression levels of NOS-2, an enzyme associated with oxidative stress. The results clearly indicated the potent antioxidant property of PL02, surpassing that of indomethacin. This enhanced antioxidant effect can be attributed to the synergistic actions of the diverse polyphenols and catechins present in PL02.

The degradation of articular cartilage and bone matrix components is a characteristic feature of osteoarthritis (OA). Early events involve the loss of type II collagen and the proteoglycan aggrecan, leading to structural and functional deterioration of the cartilage. MIA-induced OA is characterized by significant cartilage destruction, with a single injection causing over 33% loss of cartilage tissue. A major challenge in modern medicine is to prevent or regenerate cartilage to improve joint mechanical properties. Fortunately, various secondary metabolites derived from plants have shown promise in halting cartilage tissue damage (17).

For instance, the flavonoid Diosmin, found in citrus fruits, has been shown to upregulate type II collagen, the main component of the cartilage extracellular matrix. Diosmin has also been associated with increased activity of the chondrocyte-specific differentiation marker SOX-9. Clinical trials are even underway to investigate its analgesic properties (42, 57). Matrix metalloproteinases (MMPs) play a crucial role in cartilage degradation, with MMP-1, MMP-3, and MMP-13 being involved in this process. Clinical data have shown that OA patients with articular cartilage destruction exhibit high expression of MMP-13, suggesting its association with cartilage degradation (58). This finding was further supported by studies on MMP13 transgenic mice, which displayed a spontaneous OA-like phenotype characterized by articular cartilage destruction (59). Similarly, in our study, we observed increased MMP13 expression and decreased levels of aggrecan and collagen II, the main components of the extracellular matrix, indicating ongoing cartilage damage in MIA-induced osteoarthritis.

Following treatment with PL02, there was a significant decrease in MMP13 expression and a significant increase in levels of aggrecan and collagen II. As PL02 contains abundant quantities of these beneficial secondary metabolites, we observed a substantial increase in the expression of SOX-9 (*p* < 0.0001) and aggrecan (*p* < 0.001), along with a significant decrease in MMP-13 (*p* < 0.01) in the PL02-treated group compared to the disease control. These findings provide essential evidence supporting the disease-modifying potential of PL02 in protecting against cartilage degradation following MIA injection in rats. Moreover, the histopathological analysis further supports our claims.

Histological analysis of joint sections stained with H&E and toluidine blue revealed that the control group exhibited normal, healthy cartilage with a well-distributed population of chondrocytes within the extracellular matrix (ECM). In contrast, MIA injection resulted in proteoglycan loss, diminished viable chondrocytes, and disrupted proliferation zones (60). However, the PL02-treated group exhibited healthy cartilage with a normal distribution of chondrocytes within the ECM, which was rich in proteoglycans and glycosaminoglycans. This observation confirmed the protective effect of PL02 at the tissue level.

The ECM plays a vital role in providing structural and biochemical support to neighbouring cells in cartilage, serving various functions in chondrogenesis (6). It is widely known that the survival of chondrocytes is dependent on their anchorage to the ECM. Therefore, cartilage degradation and chondrocyte apoptosis are closely interconnected. The apoptotic loss of chondrocytes and the disruption of ECM integrity undoubtedly compromise cartilage structure (61).

In our study, we observed an increase in the expression of the anti-apoptotic protein BCL-2 following PL02 treatment. This finding suggests that PL02 may suppress chondrocyte apoptosis by inhibiting inflammation-induced ECM degradation. As a result, both macroscopic and histological examinations provided evidence that oral administration of PL02 effectively protected cartilage from destruction.

The subchondral bone serves as both a supplier of nutrients and a mediator of physiological and non-physiological shock absorption, providing support to the overlying cartilage (62). Any disruptions to bone cell metabolism, architecture, or structural integrity can render the bone more vulnerable to abnormal loading and may even induce abnormal responses to normal physiological loads. The structural changes observed in subchondral bone in osteoarthritis (OA) have long been considered adaptations to biomechanical alterations in articular cartilage (63, 64).

Recent pre-clinical and clinical studies have revealed that changes in bone structure may precede and contribute to cartilage pathology, and that OA progression is associated with temporal changes in bone structure (16–19). Early OA is characterized by accelerated bone turnover, resulting in thinning of the bone plate and increased porosity in the subchondral bone. Conversely, the trabecular compartment exhibits increased spacing between trabeculae and decreased bone volume fraction. As OA progresses, subchondral bone plate thickening, increased trabecular thickness, and elevated bone volume fraction are observed (16, 63).

Therefore, it was essential to investigate the effects of PL02 on the microarchitecture of the subchondral bone in the femur and tibial head at the knee joint. Our observations revealed more pronounced changes in the tibia compared to the femur, with significant differences observed in the medial compartment compared to the lateral compartment in the tibia. Detailed analysis demonstrated a significant increase in bone sclerosis, plate thickness, porosity, bone volume fraction, and trabecular thickness, along with a decrease in trabecular spacing in the MIA-induced OA group. This confirms that the MIA-induced model used in our study accurately mimics the pathological progression observed in human OA in the tibial subchondral bone, consistent with findings reported by Mohan et al. (16).

Interestingly, treatment with PL02 prevented disease progression in the subchondral bone. No thickening or sclerosis of the subchondral plate was observed.

While this study presents promising findings, it’s crucial to acknowledge several technical limitations that could have influenced the outcomes. Firstly, our approach involved analyzing joint tissue for gene expression to correlate with serum data. We aimed to comprehensively assess the anti-inflammatory and analgesic effects of PL02 by combining serum markers, behavioral observations, and gene expression levels. Additionally, lipid peroxidation is an established mechanism of cellular/tissue injury across plants and animals, serving as an oxidative stress indicator (65). Our objective was to quantify PL02’s impact within the context of MIA-induced OA, focusing on serum and gene-level evaluations, primarily related to ROS activity and its connection to extracellular matrix degradation. A third limitation pertains to the MIA-induced osteoarthritis animal model’s scope. While valuable for certain aspects of osteoarthritis research, this model primarily mimics the biochemical changes tied to osteoarthritis by inducing cartilage degradation through MIA injection. However, this approach may not fully replicate the intricate multifactorial nature of human osteoarthritis, which involves intricate interactions between tissues, joint mechanics, and systemic factors. Moreover, the model’s acute nature might not accurately mirror the gradual progression of human osteoarthritis over time. Furthermore, the MIA model predominantly affects weight-bearing joints, possibly limiting its relevance to other joint types (66). Despite these limitations, the MIA-induced osteoarthritis model remains invaluable for investigating specific facets of the disease’s pathophysiology and testing potential interventions. Integrating this model with other approaches could provide a more holistic comprehension of osteoarthritis and its potential treatments.

## 5 Conclusion

The understanding of osteoarthritis (OA) has evolved to recognize it as a complex disease involving multiple components of the joint, beyond just the cartilage or synovium. This multifaceted nature of OA makes it a highly heterogeneous condition, and therapies targeting a single tissue or aspect of the joint may not be sufficient.

**Figure 8:**
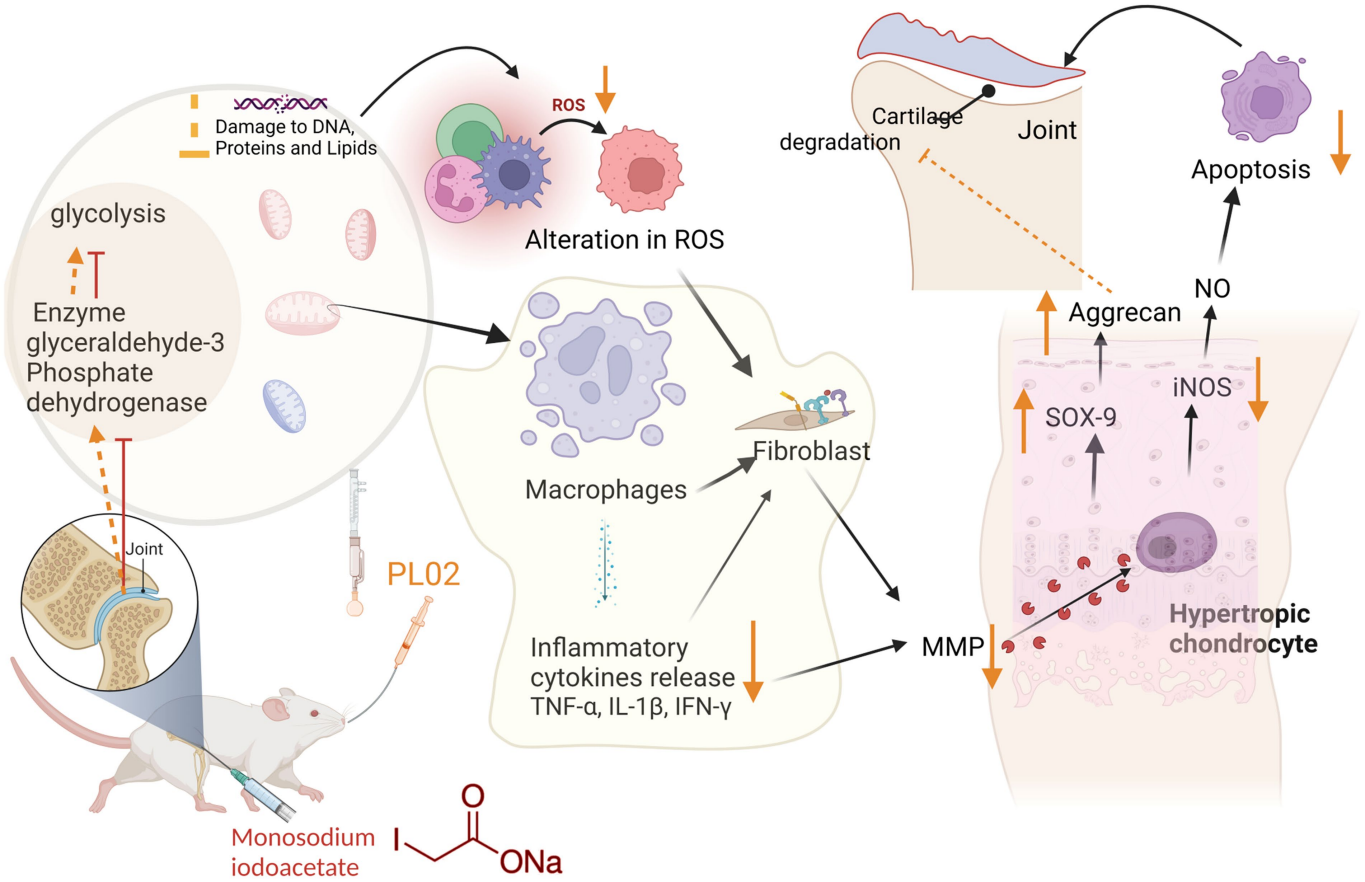
Mechanistic pathway of PL02 in treatment and management of OA.

In this context, PL02 emerges as a promising intervention. It effectively reduces pain and inflammation while providing protection against cartilage damage. Furthermore, PL02 addresses crucial aspects of the disease by preventing chondrocyte apoptosis and regulating subchondral bone turnover, as depicted in Figure 8.

The results of our study indicate that PL02 significantly modifies the progression of OA, offering potential benefits in preventing the need for joint replacement, even in advanced stages of the disease. This novel approach recognizes the complexity of OA and highlights the importance of comprehensive therapeutic strategies for its management.

## Data availability statement

The original contributions presented in the study are included in the article/Supplementary material, further inquiries can be directed to the corresponding author.

## Ethics statement

All animal studies were approved by the Institutional Animal Ethics Committee (IAEC#576/21) of the National Institute of Immunology and conducted in accordance with the ARRIVE (Animal Research: Reporting of In vivo Experiments) guidelines. The study was conducted in accordance with the local legislation and institutional requirements.

## Author contributions

PU and SG identified the ingredients for the formulation, designed the research, analyzed the final data, and wrote the manuscript. PU, AN, and DK conducted the majority of the experiments, including articular injections, collection of animal samples, molecular studies, and data analysis. AN and DK performed bone scanning using μCT and analyzed the data under the supervision of SG. VA facilitated the purchase of the formulation for commercialization, provided assistance with label claims, and protected it with a trademark. All authors contributed to the article and approved the submitted version.

## Footnotes

^1^ http://spectra.psc.riken.jp/
