# Supplementary material for: Polyherbal formulation PL02 alleviates pain, inflammation, and subchondral bone deterioration in an osteoarthritis rodent model
Source: Front Nutr. 2023 Nov 16;10:1217051. doi: 10.3389/fnut.2023.1217051 (PMC10693428; doi:10.3389/fnut.2023.1217051)
Supplement: Supplementary file 1 [file Data_Sheet_1.docx]

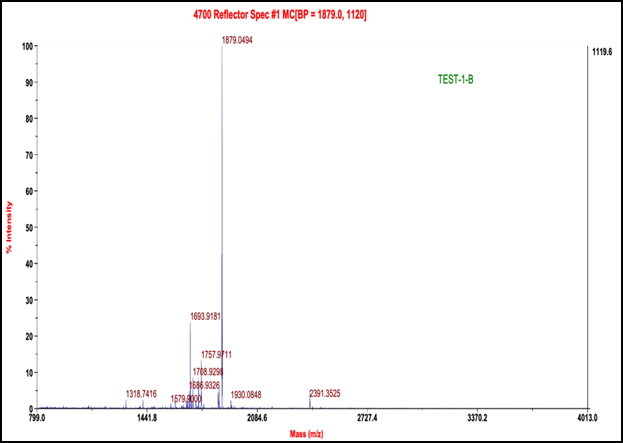


**Figure S1:** Image illustrated the MALDI-TOF mass spectrum collagen peptide to validate the molecular weight (~2 kDa)

**Table S1: Behavioral responses and general appearance of Mice/Rat treated in the sub-acute toxicity study**

| **Observation** | **Control** | **MIA** | **MIA + Indo** | **MIA + PL02** |
| --- | --- | --- | --- | --- |
| **Change In Skin** | No effect | No effect | No effect | No effect |
| **Eye color change** | No effect | No effect | No effect | No effect |
| **Food intake** | Normal | Normal | Normal | Normal |
| **General physique** | Normal | Normal | Normal | Normal |
| **Diarrhea** | Not present | Not present | Not present | Not present |
| **Coma** | Not present | Not present | Not present | Not present |
| **Drowsiness** | Not present | Not present | Not present | Not present |
| **Breathing difficulty** | Not observed | Not observed | Not observed | Not observed |
| **Sedation** | No effect | No effect | No effect | No effect |
| **Tremor** | Not present | Not present | Not present | Not present |


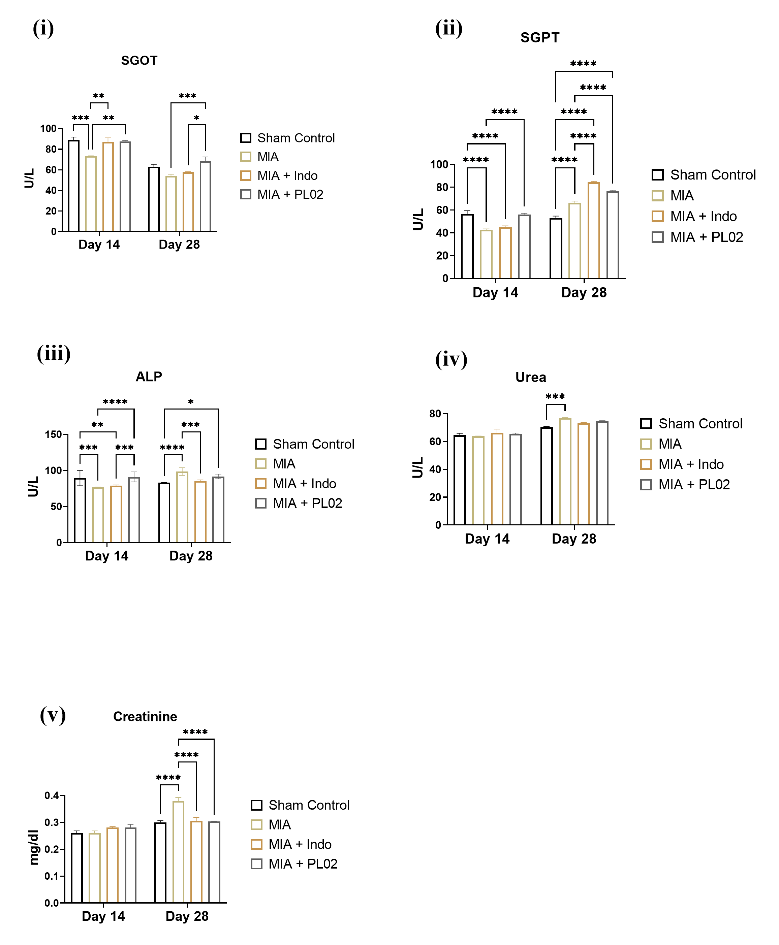

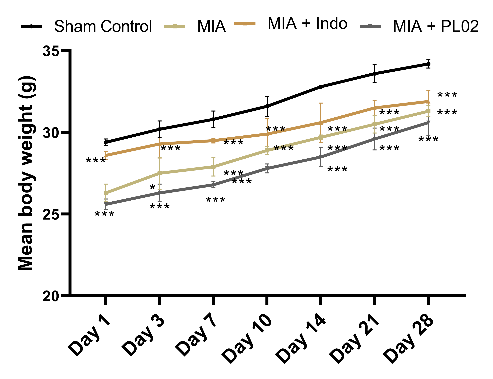


B

A

**Figure S2: PL02 showed no toxicity after 28 days of treatment in mice (A)** Average body weight recorded on days 1, 3, 7,10, 14,21, and 28 (n=8) **(B)** Serum parameter **(i)** SGOT level measured from blood serum at day 14 and day 28 (n=6) **(ii)** SGPT measured from blood serum at day 14 and day 28 (n=6) **(iii)** ALP measured from blood serum at day 14 and day 18 (n=6) **(iv)** Urea measured from blood serum at day 14 and day 28 (n=6) **(v)** Creatinine measured from blood serum at day 14 and day 28 (n=6). Statistical analysis was performed using one-way ANOVA with the Bonferroni post hoc test. Data presented as means ± SEM. *P < 0.05, **P < 0.01 and ***P< 0.001


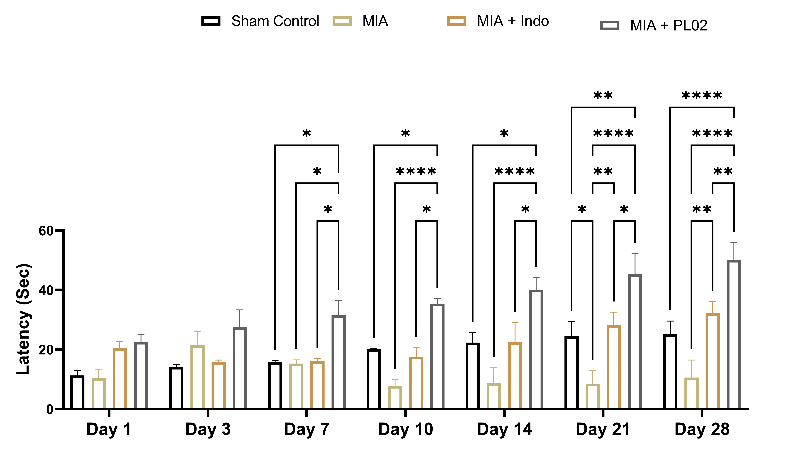

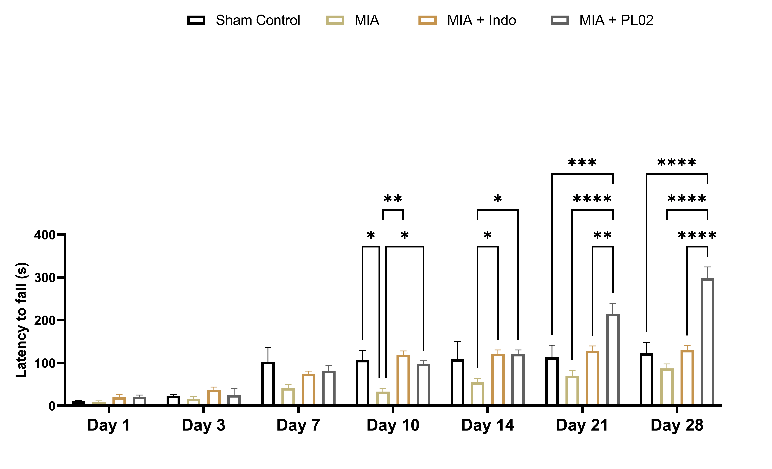


C

B

A


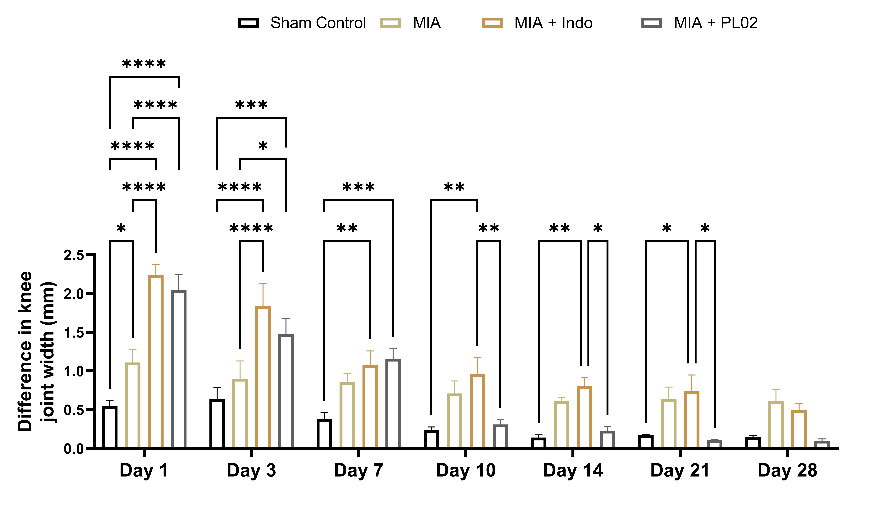

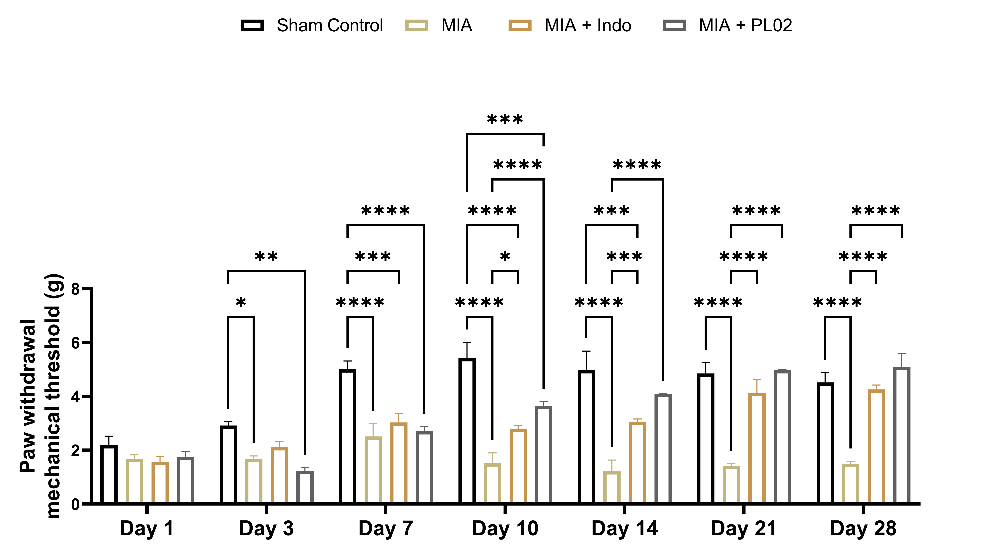


D

**Figure S3: PL02 helps in reducing the pain and inflammation (A)** Hot plate used to measure the Pain sensation on days 1, 3, 7, 10, 14, 21, and 28 (n=8) **(B)** Rota rod used to measure the joint pain through recorded the latency of fall time at days at day 1, 3, 7, 10, 14, 21 and 28 (n=8) **(C)** vernier caliper used to the measure the inflammation on knee joint at days 1, 3, 7, 10, 14, 21 and 28 (n=8) **(D)** Von Frey was performed for mechanical allodynia to measure the pain at day 1, 3, 7, 10, 14, 21 and 28 (n=8) Statistical analysis was performed using Two-way ANOVA with Bonferroni post hoc test. Data presented as means ± SEM. *P < 0.05, **P < 0.01 and ***P< 0.001


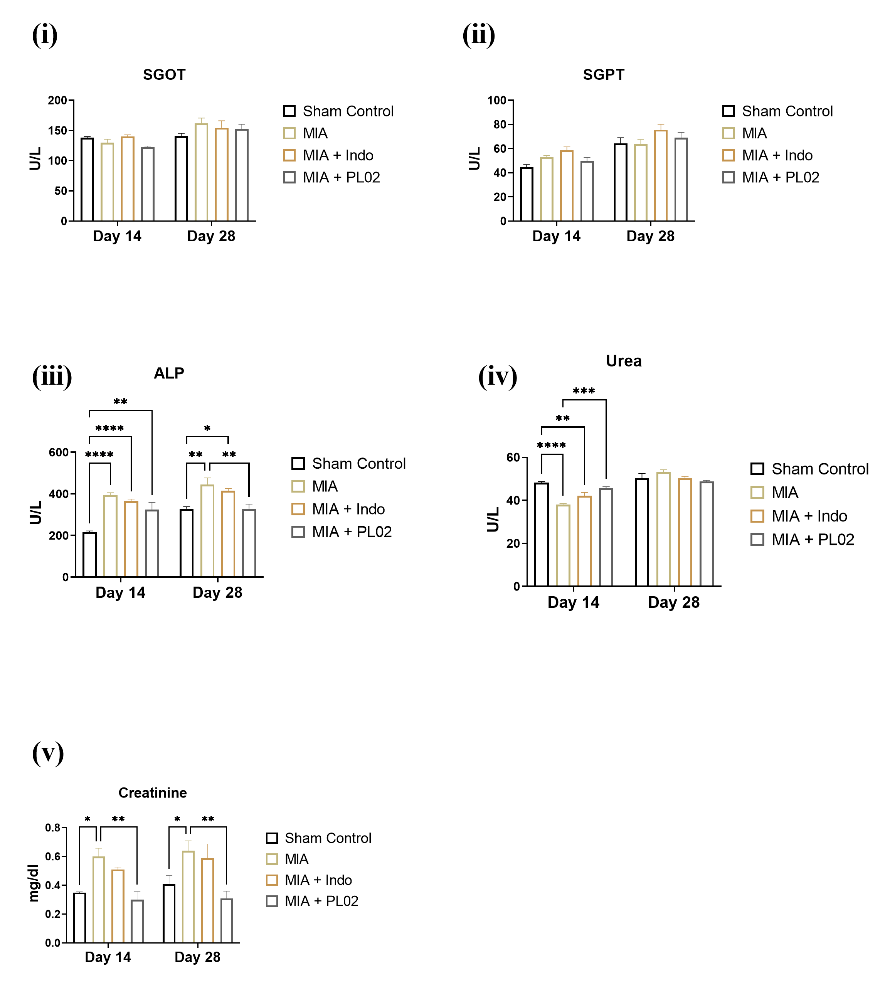

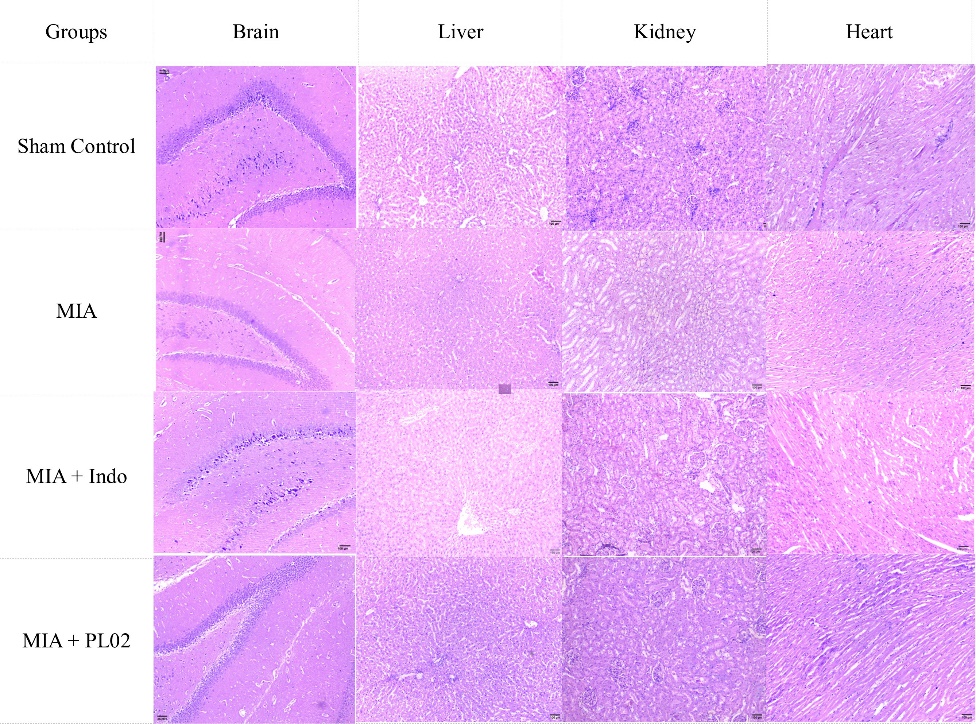


B

A

**Figure S4: PL02 showed no toxicity after 28 days of treatment**

**(A)** Serum parameter

**(i)** SGOT level measured from blood serum on day 14 and day 18 (n=6)

**(ii)** SGPT measured from blood serum on day 14 and day 28 (n=6)

**(iii)** ALP measured from blood serum on day 14 and day 28 (n=6)

**(iv)** Urea measured from blood serum on day 14 and day 28 (n=6)

**(v)** Creatinine measured from blood serum on day 14 and day 28 (n=6)

**(B)** H & E stain of the isolated vital organ after 28 days of treatment at 10 X (Brain, Liver, Kidney & Heart) scale bar 100 µm

 Statistical analysis was performed using Two-way ANOVA with the Bonferroni post hoc test. Data presented as means ± SEM. *P < 0.05, **P<0.01, ***P<0.001

**Vonfrey allodynia experiment with or without collagen**

The formulation PL02 we have developed is an orally active nutraceutical for the prevention and treatment of non-traumatic OA and related pain and inflammation symptoms. We have used hydrolyzed and purified collagen having small peptides <2kda. It is well established by various research groups that collagen peptides <2kda can be absorbed and have positive effects on joint health. Many such collagen supplements are commercially available all over the world, though its therapeutic efficacy has not been proven. We have used collagen from a nutritional point of view only. Indeed, we performed initial experiments where formulation was used with and without collagen. The results were better with collagen peptides. However, collagen alone at a given dose did not show any significant efficacy during the study period. This indicates the synergistic effect of collagen in the developed formulation PL02. As this was part of our formulation design, we did not include it in the main manuscript. See the data below plotted for 28 days.

***
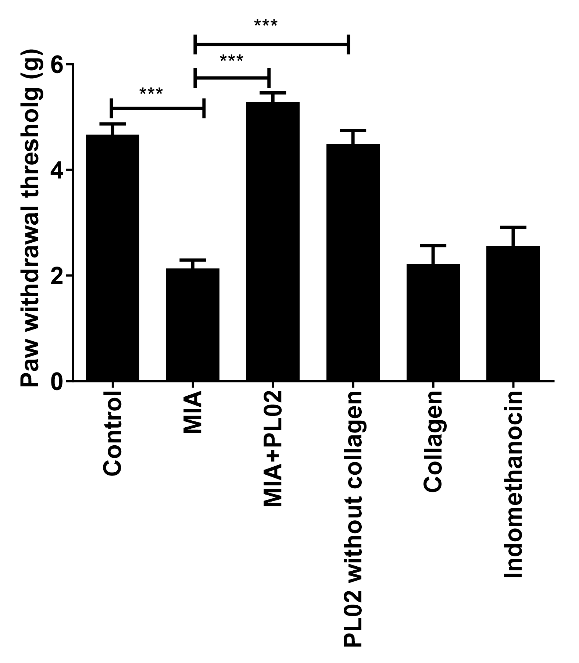
***

**Figure S5:** Von frey was performed for mechanical allodynia to measure the pain at day 28 (n=8). Statistical analysis was performed using Two-way ANOVA with Bonferroni post hoc test. Data presented as means ± SEM. *P < 0.05, **P < 0.01 and ***P< 0.001.

| **Marker** | **Forward** | **Reverse** | **Product length (bp)** | **NCBI Reference Sequence** |
| --- | --- | --- | --- | --- |
| Aggrecan | ACAGACACCCCTACCCTTGC | ACAAGGGCTTGAGAGGCACT | 90 | XM_039101034.1 |
| SOX-9 | CCTCGGGCATGAGTGAGGTG | TCCCAGCTTGCACGTCTGTT | 102 | NM_080403.2 |
| MMP13 | CTGGTCTTCTGGCACACGCT | GAGTGGTCCAGACCGAGGGA | 154 | NM_133530.1 |
| IL-1β | ACCTATGTCTTGCCCGTGGAG | GCAGGTCGTCATCATCCCAC | 110 | NM_031512.2 |
| NF-κB p65 | GGATGACAGAGGCGTGTATAAG | CCTTCTCTCTGTCTGTGAGTTG | 114 | [NM_001276711.2](https://www.ncbi.nlm.nih.gov/entrez/viewer.fcgi?db=nucleotide&id=-1879324137) |
| IL-4 | GGCAACAAGGAACACCACGG | CGTGGACTCATTCACGGTGC | 190 | NM_201270.1 |
| Bcl2 | TACCTGAACCGGCATCTGCAC | GCATGCTGGGGCCATATAGT | 82 | NM_016993.2 |
| TNF-α | GCCCTGGTATGAGCCCATGT | CCAAAGTAGACCTGCCCGGA | 123 | NM_012675.3 |
| CXCL-12 | CCTCAACACTCCAAACTGTGCC | ACGGAGGTCAGCCTTCCTCA | 178 | NM_022177.3 |
| Ccl2 | GCAGGTCTCTGTCACGCTTC | AGCAGGTGAGTGGGGCATTA | 101 | NM_031530.1 |
| TIMP 2 | CGGACTCCTAGAGACACGCT | TCTGCTCAGGAATTTGAAGGCAG | 70 | XM_039099407.1 |
| NOS2 | CTTGGTGAGGGGACTGGACTTT | ACCAACTCTGCTGTTCTCCGT | 104 | NM_012611.3 |
| Coll type II | CTCTGGCAAAGATGGCTCTAAT | GTCATGCTGTCTCAAGGTACTG | 244 | XM_006242308.4 |
| Ptgs2/Cox2 | TCAAGGGAGTCTGGAACATTG | GCTTCCCAACTTTTGTAACCG | 139 | NM_017232.3 |
| CGRP 1 | CCAGATCAAGAGTCACCGC | GGAGAACTTCAGAAAGCCCATG | 137 | NM_001033955.1 |
| Beta Actin | CACTTTCTACAATGAGCTGCG | CTGGATGGCTACGTACATGG | 148 | NM_031144.3 |
| GAPDH | GGTCGGTGTGAACGGATTT | TGGAAGATGGTGATGGGTTTC | 219 | NM_017008.4 |

**Table S2: List of Primers with sequence used in Quantitative Real-time PCR analysis**
